# Stability Increase of Phenolic Acid Decarboxylase by a Combination of Protein and Solvent Engineering Unlocks Applications at Elevated Temperatures

**DOI:** 10.1021/acssuschemeng.3c06513

**Published:** 2024-02-21

**Authors:** Kamela Myrtollari, Elia Calderini, Daniel Kracher, Tobias Schöngaßner, Stela Galušić, Anita Slavica, Andreas Taden, Daniel Mokos, Anna Schrüfer, Gregor Wirnsberger, Karl Gruber, Bastian Daniel, Robert Kourist

**Affiliations:** † Institute of Molecular Biotechnology, Graz University of Technology, Petersgasse 14, 8010 Graz, Austria; ‡ Austrian Centre of Industrial Biotechnology, ACIB GmbH, Petersgasse 14/1, 8010 Graz, Austria; § Adhesive Technologies, Henkel AG & Co. KGaA, Henkelstr. 67, 40191 Düsseldorf, Germany; ∥ BioTechMed-Graz, Mozartgasse 12/II, 8010 Graz, Austria; ⊥ Faculty of Food Technology and Biotechnology, Department of Biochemical Engineering, University of Zagreb, Pierottijeva 6, HR-10000 Zagreb, Croatia; # Institute of Molecular Biosciences, University of Graz, NAWI Graz, Humboldtstraße 50/3, 8010 Graz, Austria

**Keywords:** biocatalysis, enzymatic decarboxylation, biobased polymers, deep eutectic solvents, ancestral sequence reconstruction

## Abstract

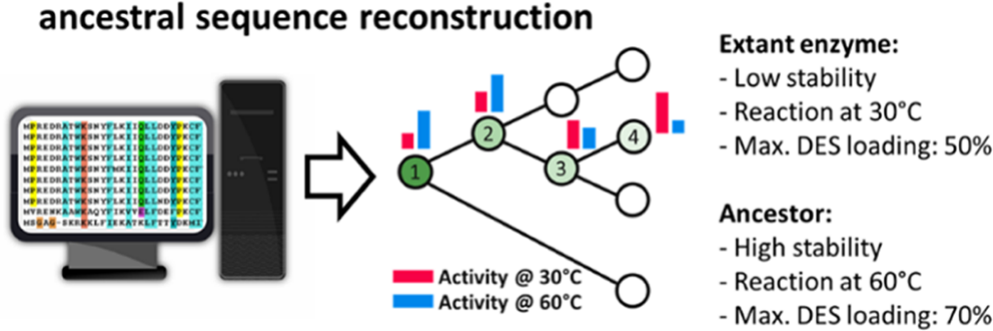

Enzymatic decarboxylation of biobased hydroxycinnamic acids gives access to phenolic styrenes for adhesive production. Phenolic acid decarboxylases are proficient enzymes that have been applied in aqueous systems, organic solvents, biphasic systems, and deep eutectic solvents, which makes stability a key feature. Stabilization of the enzyme would increase the total turnover number and thus reduce the energy consumption and waste accumulation associated with biocatalyst production. In this study, we used ancestral sequence reconstruction to generate thermostable decarboxylases. Investigation of a set of 16 ancestors resulted in the identification of a variant with an unfolding temperature of 78.1 °C and a half-life time of 45 h at 60 °C. Crystal structures were determined for three selected ancestors. Structural attributes were calculated to fit different regression models for predicting the thermal stability of variants that have not yet been experimentally explored. The models rely on hydrophobic clusters, salt bridges, hydrogen bonds, and surface properties and can identify more stable proteins out of a pool of candidates. Further stabilization was achieved by the application of mixtures of natural deep eutectic solvents and buffers. Our approach is a straightforward option for enhancing the industrial application of the decarboxylation process.

## Abstract 2

In this study, we designed a new thermostable decarboxylase that can perform reactions at elevated temperatures and in deep eutectic solvents.

## Introduction

Enzyme catalysis significantly contributes to the transition of the chemical and pharmaceutical industries toward sustainable production in a circular economy. While biocatalysis has achieved remarkable success in manufacturing fine chemicals and pharmaceutical intermediates, a current challenge is the production of specialty and bulk chemicals, which require large-scale production under mild reaction conditions. This poses demanding requirements in terms of space-time yields and biocatalyst productivity.^1^ A big challenge for many enzymatic processes is the limited solubility of hydrophobic substrates in water. In fact, one of the very first studies on the directed evolution of enzymes aimed at an increased tolerance of a carboxylesterase toward water-miscible organic solvents to allow higher substrate loadings.^2^ The substrate solubility is particularly relevant for applications that require a homogeneous solution, such as continuous flow.^3,4^

**Figure 1:**
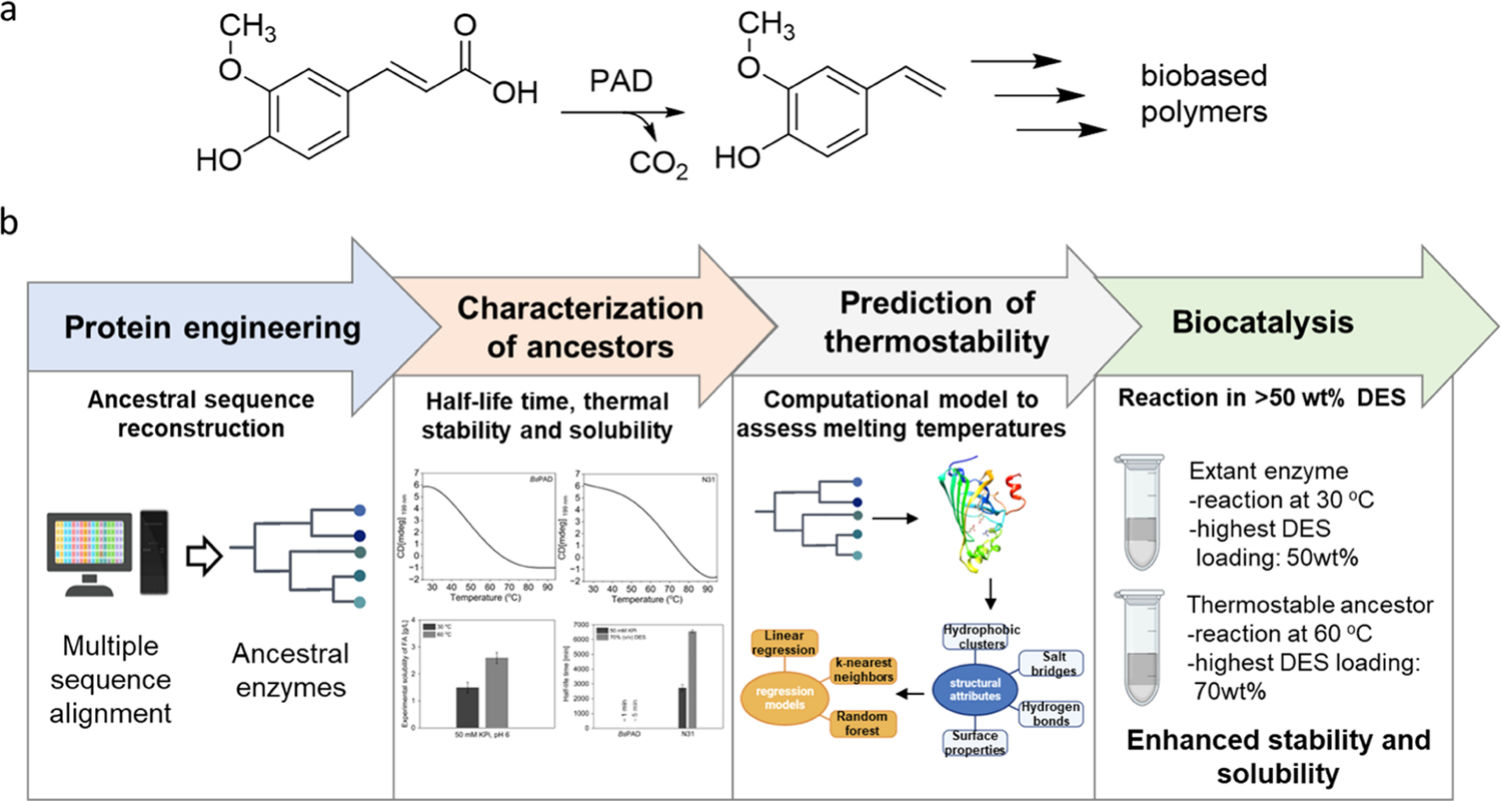
(a) Decarboxylation of ferulic acid in the presence of PAD. (b) Schematic representation of the workflow used to obtain thermostable decarboxylases.

Phenolic acid decarboxylase (PAD) has been extensively studied for the biocatalytic production of phenolic styrenes from biobased precursors (Figure 1a).^5−10^ They find application in the production of polymeric cross-linkers with direct application in adhesion technology.^11−14^ Copolymerization of the monomer with other monomers can alter the obtained polymer and further modify its properties.^15−18^ Furthermore, their dimerization gives rise to symmetric dihydroxystilbenes, which are highly potent antioxidants.^19^ To date, several PADs have been biochemically analyzed and structurally characterized.^19−22^ PADs adopt the lipocalin fold, and the core of the protein is formed by two mutually perpendicular β-sheets. In solution, a homodimer is formed by two monomers that are related by an improper 2-fold axis. This leads to a symmetric set of each 2-fold established interactions at the dimer interface (Figure S1).

A challenge faced in the industrial application of PAD is the low solubility of hydroxycinnamic acid substrates. To address this issue, various solvents were employed in combination with phenolic acid decarboxylases. Petermeier et al. recently developed a single-pot chemoenzymatic cascade by combining enzyme immobilization with medium engineering to produce styrenes in a tailor-made solvent system with controlled water activity at high volumetric productivity.^10^ In addition, deep eutectic solvents (DES) have been shown to allow a high substrate solubility.^4,5^

Recently, we developed the decarboxylation of hydroxycinnamic acids catalyzed by PAD from *Bacillus subtilis* (*Bs*PAD) in 50% (v/v) DES/H_2_O, which allowed substrate loadings of up to 300 mM, albeit with a slurry starting solution.^5^ In contrast, continuous flow setups require complete dissolution of the starting material. In aqueous monophasic solutions, Grabner et al. increased the substrate loading from 5 to 20 mM using deep eutectic solvents (DES) as a homogeneous starting solution.^4^ This report marks the highest substrate loading reported for the biocatalytic decarboxylation of hydroxycinnamic acids applied in a continuous flow setup. Biphasic systems, organic solvents, and nonconventional solvents present themselves as very promising approaches to overcome the limited substrate solution of phenolic acids. Furthermore, the substrate solubility can be further increased by applying higher reaction temperatures.

For all of these approaches, however, the stability of the enzyme is a crucial parameter. Biocatalysts should have outstanding stability to withstand long incubation times, tolerate elevated temperatures, and show high resistance toward various additives and solvents.^23^ The biocatalyst productivity determines the energy demand and waste accumulation associated with the production of the biocatalyst by cultivation in microbial production systems and, quite frequently, subsequent immobilization on a solid carrier. Any increase in stability directly reduces the effort and environmental footprint associated with enzyme production. This importance is also reflected by many studies aiming to fine-tune enzyme stability via directed evolution and protein engineering.^24−26^ As an aside, thermostability is a highly desired feature for new applications such as in three-dimensional (3D) bioprinting of biocatalytic flow reactors.^27^

Proteins of increased thermal stability may be obtained either by searching for homologues in hyperthermophiles,^28,29^ or by protein engineering.^30^ Some characterized PADs show moderate thermostability. Decarboxylation of ferulic acid at moderately higher temperatures was described by Ni et al. by using a PAD from *Bacillus coagulans*, which displays maximum activity at 50 °C.^31^ However, no PAD has been isolated yet from thermostable or even hyperthermostable organisms, leaving protein engineering as the best option to create a thermostabilized decarboxylase.

The thermostability of proteins is often increased by the stepwise addition of interactions between amino acid residues aiming to increase the rigidity of the protein. This approach is straightforward, but each successful mutant increases the stability by only a few degrees and reduces the activity considerably. As an alternative, reconstructed ancestral proteins are often more thermostable than modern descendants.^24^ Ancestral sequence reconstruction (ASR) has emerged as a useful methodology for engineering proteins with enhanced properties and boosted thermostability.^32−34^ The ancestral sequences are reconstructed by inferring a phylogenetic relationship between modern homologues and applying a statistical model of amino acid substitution to calculate sequences at internal nodes of the phylogenetic tree.^35,36^

In this study, we show that the combination of the reconstruction of decarboxylase ancestors and solvent engineering is straightforward to achieve a substantially higher thermostability, which is a prerequisite for operating at elevated temperatures and therefore increased substrate solubility. The 3D structures of the thermostable ancestral variants were solved by X-ray crystallography in order to elucidate the molecular basis of the stabilization and to generate models for the prediction of thermostability (Figure 1b).

## Results and Discussion

**Figure 2:**
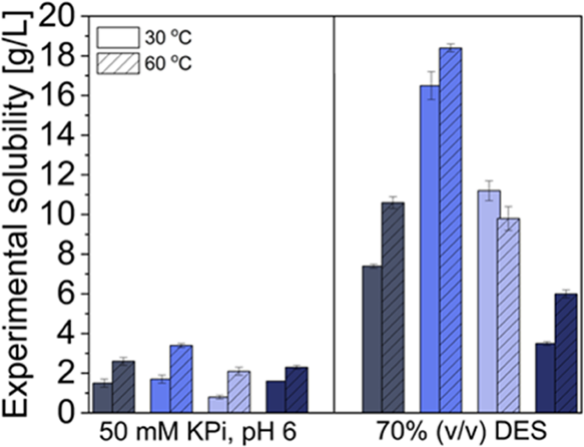
Experimentally determined solubility of ferulic acid (■), caffeic acid (dark blue box solid), coumaric acid (light blue box solid), and sinapic acid (violet box solid) in buffer and DES/buffer mixture at 30 and 60 °C. Buffer: 50 mM KPi, pH 6, DES: 1 ChCl/2 Gly (mol/mol). Experimental solubility was determined by high-performance liquid chromatography (HPLC).

To study the effect of the temperature and DES concentration on the substrate loading, we determined the experimental solubility of various hydroxycinnamic acids in buffer and DES/buffer mixtures at different temperatures (Figure 2). For example, the experimentally determined solubility of ferulic acid in 50 mM phosphate buffer, pH 6, is 1.5 g/L at 30 °C or 2.6 g/L at 60 °C (Figure 2), respectively, which is low for industrial biocatalysis. We have previously shown that the solubility can be substantially increased in choline chloride (ChCl)-based DES.^5^ DES enables substrate loadings of up to 7.4 g/L of ferulic acid in 70% (v/v) DES/buffer mixtures at 30 °C as a homogeneous solution. Increasing the temperature to 60 °C enhanced the solubility to 10.6 g/L ferulic acid. Similar results are observed for caffeic, coumaric, and sinapic acid (Figure 2).

**Figure 3:**
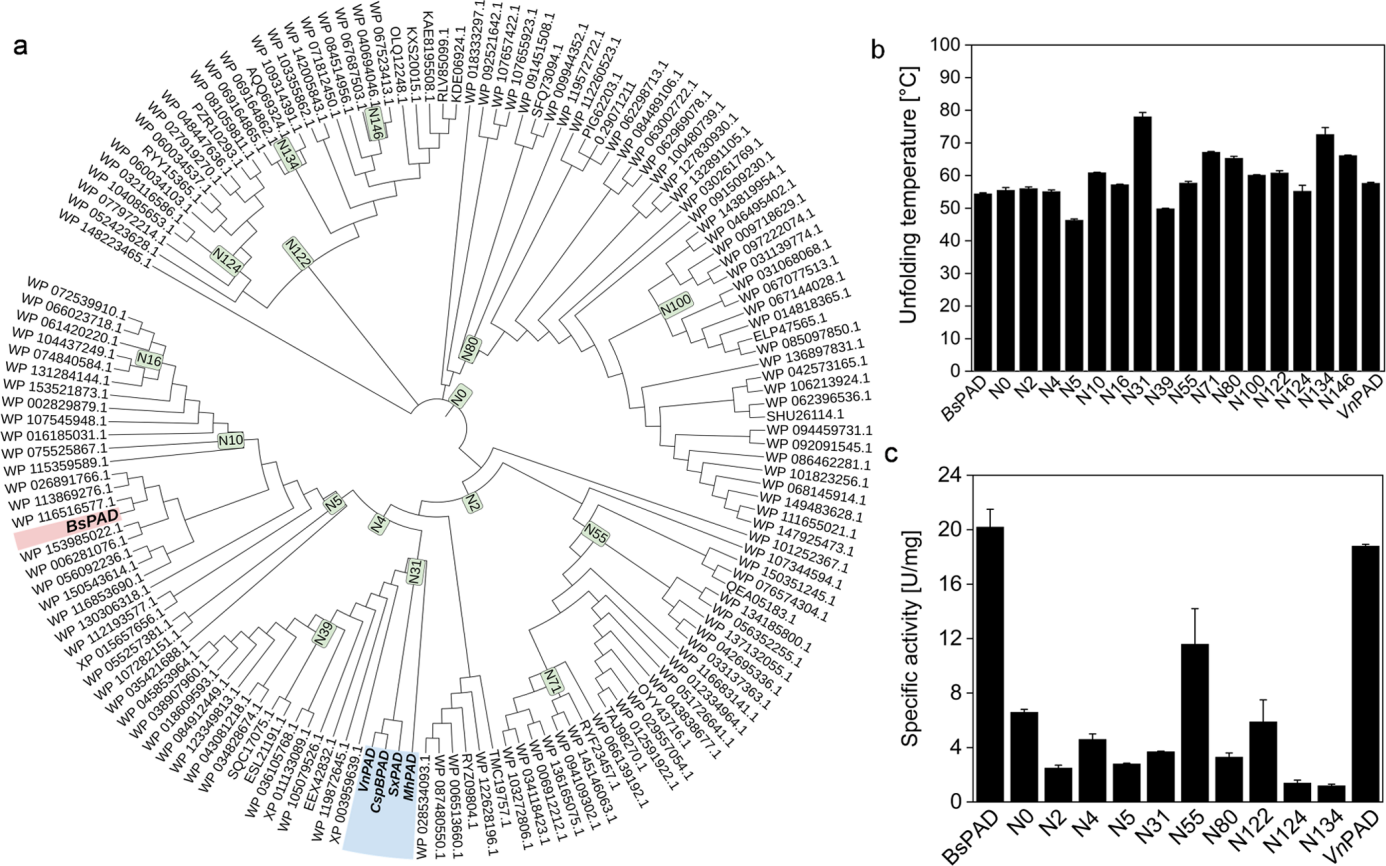
Ancestral reconstruction of PAD yields a highly thermostable ancestor. (a) Phylogenetic tree composed of 150 sequences of extant phenolic acid decarboxylases. Evolutionary distances were computed with the maximum likelihood method by using the Jones–Taylor–Thornton (JTT) matrix. The phylogenetic tree was visualized using iTOL. Ancestors selected in this study are highlighted in green. (b) The midpoint of the thermal unfolding temperature *T*_m_ of each protein was measured by circular dichroism (CD)-spectroscopy at 199 or 224 nm. Conditions: 0.1–0.2 mg/mL of purified protein dissolved in 50 mM KPi, pH 6. The ancestral protein N31 shows higher thermostability in comparison to the extant enzymes from *B. subtilis* and *Vibrio nigripulchritudo*. (c) Comparison of the specific activities of ancestors with extant *Bs*PAD toward ferulic acid. Initial reaction rates were measured by following the substrate depletion via HPLC. Reaction conditions: ferulic acid (10 mM, 5% (v/v) dimethyl sulfoxide (DMSO)), 50 mM KPi, pH 6, 2–10 μg of purified enzyme, 30 °C, 600 rpm. Data are the means and range of duplicate measurements. All data were analyzed with Origin 2022b.

To unlock the full potential of the decarboxylation reaction in DES at higher temperatures, we aimed to enhance the stability of PAD through enzyme engineering using ancestral sequence reconstruction (ASR). We collected 150 putative PAD protein sequences using a BLAST algorithm^37^ and used ASR in search of thermally stable ancestral enzymes. Ancestral sequences were reconstructed by the Maximum Likelihood (ML) method using GRASP,^38^ and the phylogeny was prepared using MEGAX.^39^ Initially, 10 different ancestral genes with a 50–70% sequence identity to *Bs*PAD were chosen for experimental verification (Figure 3a). As the precise prediction of the thermostability is not possible yet, 10 sequences appeared to be a reasonable number to obtain a representative set of other ancestors from distant clades of the phylogenetic tree, to cover a broad sequence space, and to obtain proteins with a wide spectrum of properties.

All ancestors were produced in *Escherichia coli* as active proteins with yields ranging from ∼7 to 136 mg of purified enzyme per liter of cultivation volume (Figure S2). The unfolding temperatures of all enzymes were determined by circular dichroism spectroscopy, and the activities were assayed with ferulic acid (Figure 3b,c). The relatively recent ancestor N31 has a significantly higher thermostability (*T*_m_ = 78.1 °C) than proteins from more distant clades, such as N2 (*T*_m_ = 56.1 °C) or N4 (*T*_m_ = 55.2 °C). A further look into the origin of the sequences of the descendants revealed that none of them originate from thermostable microorganisms (Table S1). As N31 is a relatively recent ancestor, we also expressed a yet uncharacterized PAD from the marine bacterium *V. nigripulchritudo* (*Vn*PAD) from the same clade.

*Vn*PAD has a substrate spectrum comparable to that of known PADs (Figure S3) and bears a typical hallmark of enzymes from mesophilic organisms (*T*_m_ = 57.7 °C). All ancestors except N5 showed an intrinsic thermostability higher than that of known extant enzymes, including *Bs*PAD (*T*_m_ 54.5 °C). The identification of ancestor N31 with a 23.6 °C higher thermostability than *Bs*PAD represents a notable advancement over the previously reported thermostable *Bc*PAD with an optimal activity at 50 °C. Although *Bc*PAD shares 79% amino acid identity with our extant *Bs*PAD, it is more stable, possibly because it originates from a thermophilic bacterium.^31^ Notably, although N31 did not evolve from thermophilic bacteria, it has a much higher thermostability and can perform decarboxylation reactions at temperatures exceeding 60 °C. We also aimed to improve the thermostability of N31 further using the publicly available PoPMuSiC server.^40^ However, all five suggested single-site variants reduced the thermostability of N31 (Table S2).

**Figure 4:**
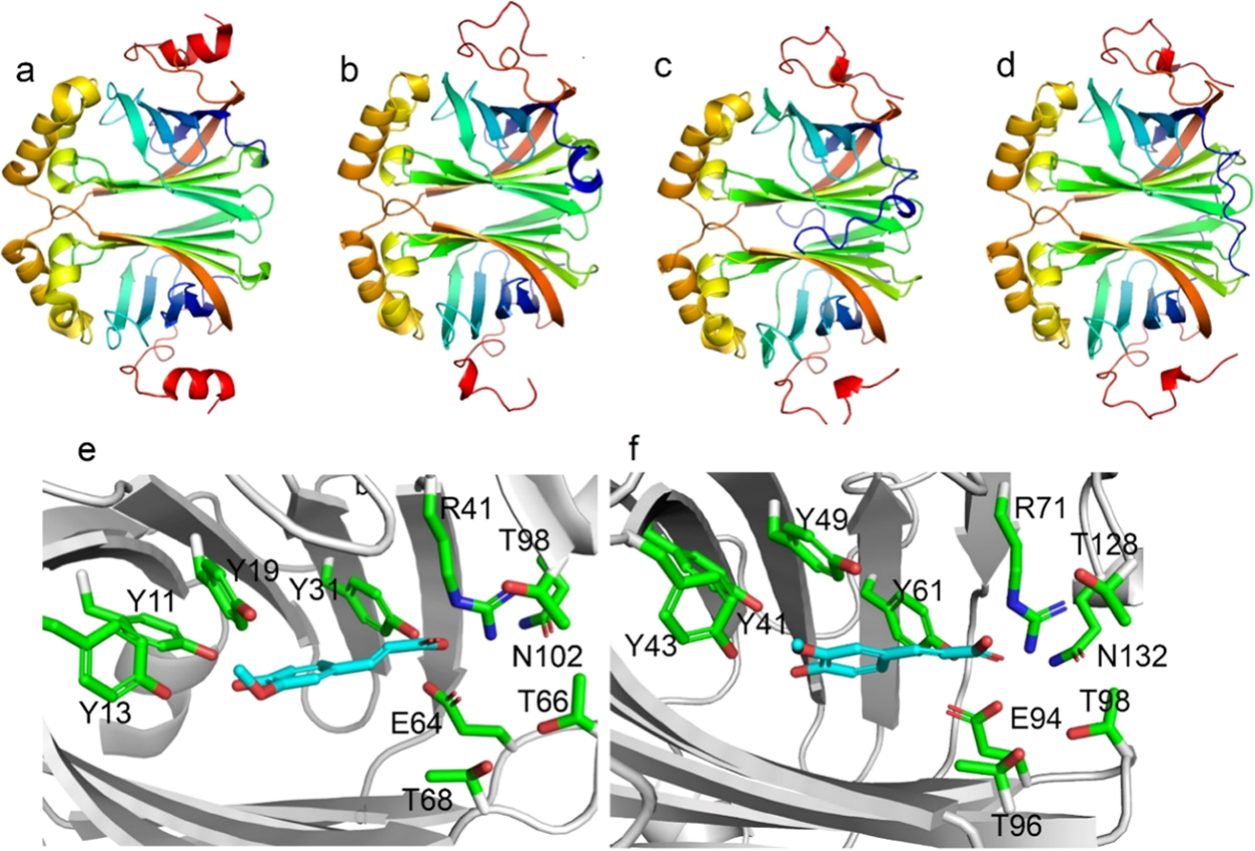
Homodimer of (a) *Bs*PAD, (b) N31, (c) N55, and (d) N134. (e) Structure of *Bs*PAD docked with ferulic acid. (f) Structure of N134 docked with ferulic acid. The structures are visualized with PyMOL.

At present, there is no clear understanding of the origins of the thermostability of ancestral PADs. To gain a better understanding based on their activity and stability, we determined the structures of N31, N55, and N134 via X-ray crystallography. The models for the remaining ancestors were predicted using AlphaFold 2.^41^ N31, N55, and N134 were crystallized with one, two, and three homodimers in the asymmetric unit at a resolution of 3.1, 1.6, and 2.5 Å, respectively. For a given PAD, the monomers were found to be highly similar to a root-mean-square deviation (RMSD) of below 0.3 if aligned. Also, the overall structures of the PADs were found to be conserved, leading to an RMSD below 1 Å if aligned to *Bs*PAD. The highest structural divergence was found in the C- and N-terminal regions. The respective homodimers of *Bs*PAD (PDB 2P8G) and the ancestral decarboxylases N31 (PDB 8B30), N55 (PDB 8ADX), and N134 (PDB 8A85) are depicted as a cartoon in Figure 4a–d with the individual PAD monomers colored from the N-terminus to the C-terminus from blue to red.

Additionally, the two β sheets form a dimerization interface that is highly conserved in all structures. The entrance to the active site was suggested to be regulated by a loop between β1 and β2. The different conformations of this loop suggest an opening and closing of the active site via this loop.^20^ The C-terminal part (depicted in red) shows some structural and sequential diversity. N55 and N134 harbor an N-terminal loop (depicted in blue) absent in N31 and *Bs*PAD that contributes to some extent to the dimerization (compare also Figure S4). All ancestors showed a highly conserved active site architecture with identical catalytic machinery (Figures 4e,f and S5).

Despite the overall similarity of the active sites, the ancestors show reduced activity compared to *Bs*PAD at moderate temperatures. Two distinct sets of residues were postulated to participate in the decarboxylation of *p*-hydroxycinnamic acids.^42^ Wuensch et al. postulated a productive binding mode in which the *p*-hydroxy group interacts with Tyr11 and Tyr13, and the carboxylic acid interacts with Arg41, which facilitates the decarboxylation concertedly with Glu64, Thr68 Thr66, and Tyr31.^43^ The molecular docking of ferulic acid revealed the same productive binding mode for all PADs (Figures 4e,f and S6). This is in good agreement with their common activities and the enzymatic mechanism that was postulated for this enzyme class.

While ancestral reconstruction is a useful tool for enzyme stabilization, predicting the stability of different ancestral sequences is still challenging. A reasonable assumption links higher conformational flexibility to reduced stability and vice versa.^44^ To assess their dynamic properties, molecular dynamics (MD) simulations were set up for individual PADs. Root-mean-square fluctuation (RMSF) plots of the C-alpha atoms differed qualitatively but did not indicate statistically significant correlations among the mean RMSF values, their variances, or standard deviations with their thermal stability in all investigated structures. For all models, a similar distribution of flexibility was observed if plotted on their sequence (Figure S7) or structure (Figure S8).

**Figure 5:**
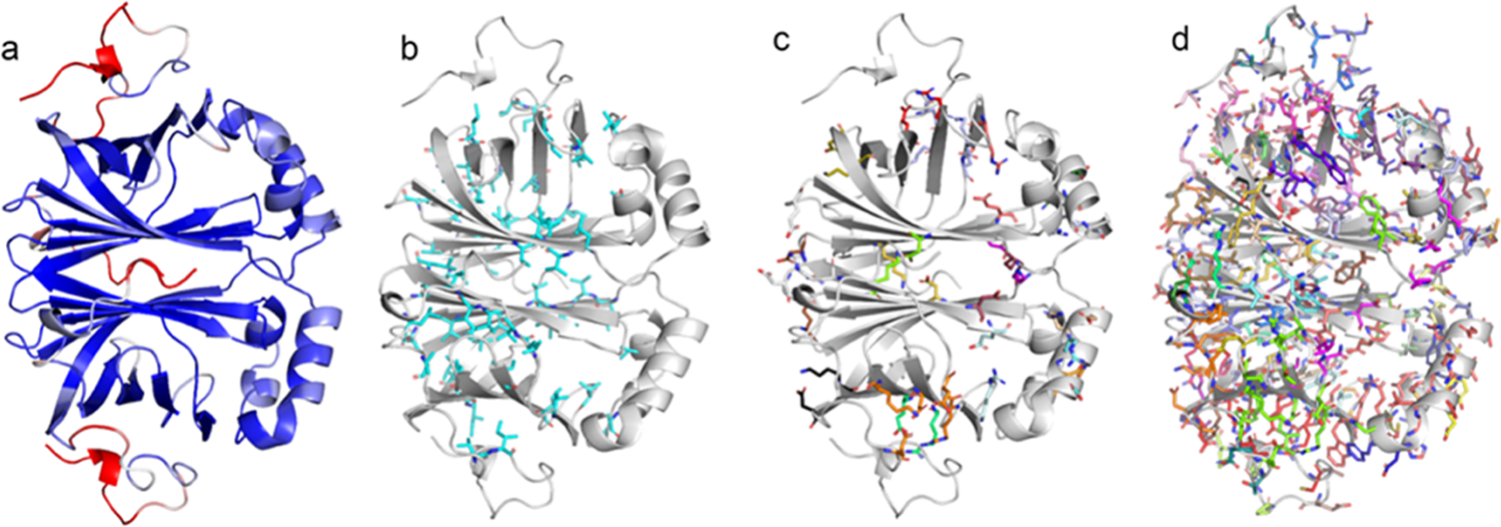
Analysis of the structural properties of PADs with N55 as an example employing MD simulation-derived data and the pipeline-generated PyMOL-executable scripts. (a) N55 depicted as a cartoon and colored by RMSF. (b) Residues in hydrophobic clusters. (c) Residues involved in salt bridges. (d) Residues involved in hydrogen bonds. All structures are visualized using PyMOL.

The lowest flexibility of the PADs was observed in the β sheets that form the core of the enzyme (compare Figure S1a highlighted in blue). A moderate increase in flexibility can be observed for the loops from β1 to β2 and β3 to β4 (Figures 5a and S9). The respective movement has previously been reported to putatively open and close the active site. Also, α helices 10–13 (compare Figure S1a, underlaid in red) show higher flexibility than the core (compare Figure S9). The residues corresponding to N132 and T41 in N55 originate from these helices. Both were found to form a hydrogen bond to the NH1 atom of the catalytically active arginine in all crystal structures. This bidentate complexation of a catalytic residue putatively affects its position, thereby determining the catalytic properties of the enzyme (Figures S9 and S10). The highest flexibility can be found in the N-terminal loop Ala24 to Val37 and the C-terminal extension Cys168 to Asn192 covering the core β-sheet (compare Figure 5a and Figure S8).

**Figure 6:**
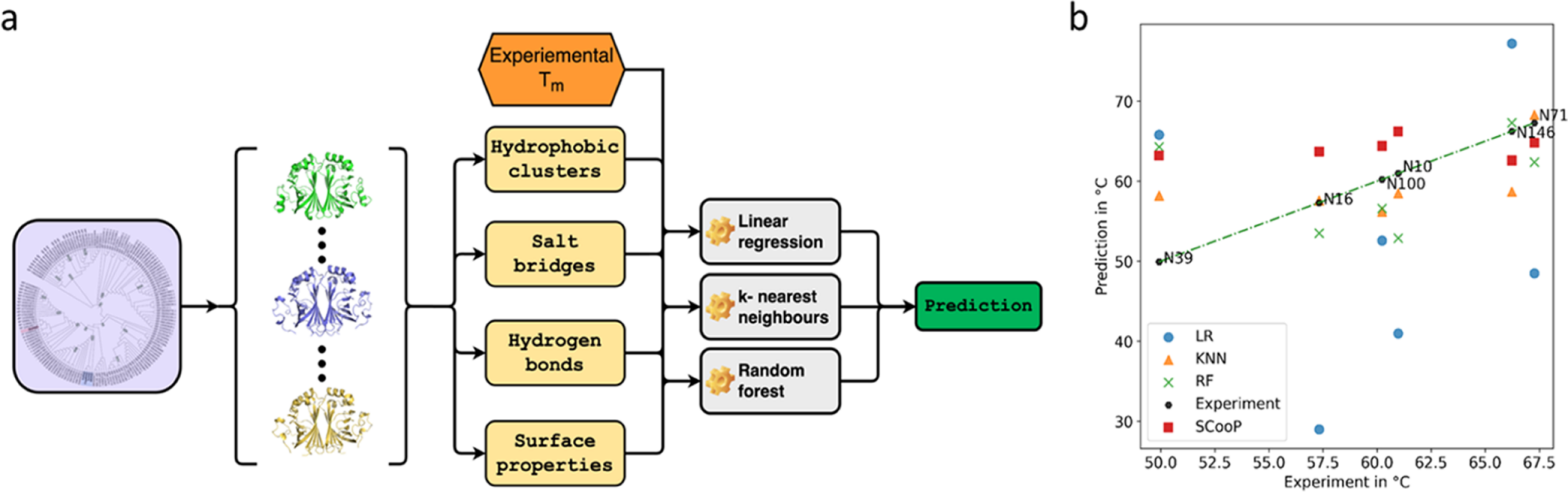
(a) Overview of the model training and prediction workflow: From the ancestral sequence reconstruction, sequences are selected and expressed, and the experimental *T*_m_ is determined. In parallel, AlphaFold 2 models are built from these protein sequences, various structural attributes are calculated, and using these and the experimental *T*_m_ as a target, various regression models are fitted to this data. These models can then be used to predict the unfolding temperature of untested ancestors, and these predictions can be used to decide which ancestor to test and which not to test in the lab due to their low predicted *T*_m_. (b) PAD unfolding temperatures: Experimental and predicted values of the three best-performing models and the SCooP server. LR: linear regression, KNN: k-nearest neighbors’, RF: random forest.

To elucidate the structural basis of the differences in thermostability, we have programmed a pipeline that calculates structure-determining attributes such as salt bridges, hydrophobic interactions, and hydrogen bonds as well as protein surface properties (Figure 6a). Additionally, it creates PyMOL-executable scripts that select the respective residues that take part in the formation of a given attribute in the protein structure and present them as sticks (Figure 5). The benchmark for the functionality of the pipeline and the quality of the models was the ability to identify the same set of interactions in the individual monomers of a given dimer. Additionally, due to the nature of the dimer and the interface β5–β6–β7–β8–β9, the arrangement of the residues contributing to the respective attribute must be symmetrical. This was verified via visual inspection using a pipeline-generated set of PyMOL scripts (compare Figures S1 and 5). Statistics are applied to these attributes, which transforms them into a suitable form to fit different regression models. The respective trained algorithms can make predictions about other untested variants (Supporting Information (SI) section Network Analysis).

The analysis of the structures, as represented in Figure 5, indicates that the core formed by the β-strands was not only found to exhibit the lowest RMSF values but also mainly populated by hydrophobic residues (compare Figure 5a,b). Salt bridges are largely absent from the core except for the catalytic Arg73 and Glu96. Salt bridges are more prominent at the interface between both monomers and between the β-sheet β9 β1 β2 β3 β4 and the C-terminal region that shields the sheet from the solvent (Figure 5c). H-bonds are distributed evenly throughout the whole protein (Figure 5d). With the pipeline, it is possible to extract five structural attributes (hydrophobic clusters and two surface properties) that show statistically significant (*p* < 0.05) linear correlation with the measured thermostability (compare Table S3). Although the attributes alone do not have sufficient predictive power, they can be used in various regression models to predict *T*_m_ values for untested ancestors of the same enzyme family. To achieve the best prediction, all possible 4-fold combinations of all single attributes of Table S4 were tested by running a multiple linear regression, a random forest regression, and a k-nearest neighbors (KNN) regression for each using leave-one-out to determine the mean absolute error (MAE). To avoid overfitting, the number of tested parameters was fixed to four. The best-performing models were then fitted to the full training data of 10 ancestors and 2 extant enzymes. Subsequently, a test data set of six randomly selected ancestors, not used in the fitting process, was used to assess the performance of the models and test their applicability in predicting the *T*_m_ and selecting more stable proteins. Although the linear regression performed best when using leave-one-out evaluation on the training data set, it showed the lowest performance of all models on the test data set, indicating a lower ability to extrapolate to unseen data. On the other hand, KNN and random forest performed similarly in training and testing (Table 1).

**Table 1:** Highest Ranked Regression Models with 4-Fold Combinations for the Regression Methods: Linear, Random Forest, and k-Nearest Neighbors^a^

regression method		combination				MAE in K (fit/test)	recall
linear	attribute	MEAN CA	MEAN CC	neg HYDROPATHY	MEAN NWS HB	3.3/16.9	1
	coefficient	41.24	38.24	5.16	4.66		
random forest	attribute	MAX CA	SUM NWS HB	MAX NWS SB	MAX IA SB	4.9/6.0	2
	feature importance	0.56	0.20	0.15	0.08		
k-nearest neighbors	attribute	MAX IA SB	MAX CA	SUM CHARGE	MAX NWS SB	4.2/3.9	3

a Listed are the respective attributes with their predictive power (linear: coefficient, random forest: feature importance, and k-nearest neighbors: not applicable). The models are ranked according to the recall from low to high, and the attributes according to their predictive power (high to low, left to right). The MAE shows the leave-one-out MAE for the fit on the training data set and the MAE for the predictions on the test data set. Recall shows how many of the three proteins with the highest *T*_m_ were correctly identified when the proteins were sorted by predicted *T*_m_.

When asked to recall the three proteins with the highest unfolding temperatures, the KNN model can successfully recall the three proteins with the highest unfolding temperatures out of the six proteins in the test data set. When comparing the attributes used by the models to achieve their best performance, no clear conclusions can be drawn as to which attributes are the main drivers of improved thermal stability. We compared the performance to other protein structure-based *T*_m_ prediction tools, SCooP^45^ and DeepSTABp^46^ (Table S5).

The main features it considers are the number of residues, the surface accessible side chain area, the host organism’s environment temperature, and temperature-specific statistical potentials. This model reaches a mean absolute error (MAE) of 8.3 K on the training set and 5.9 K on the test data set and recalled two of the three proteins with the highest *T*_m_ correctly, indicating that our best model outperforms the current state-of-the-art approach. Our models are trained on data similar to the test data used. SCooP, on the other hand, is a global model that can be used for any family of proteins.

This gives our models an initial advantage in this comparison. The predictions for the individual PADs of the test data set compared to those of the experimental *T*_m_ are summarized in Figure 6b. Due to the limited training data, these models are best at finding potential candidates with a higher unfolding point and, therefore, are better at selecting candidates to test in the laboratory rather than determining the exact unfolding temperature. This can potentially reduce the workload required in the lab, saving resources and time. Here, we show the feasibility of this approach that has not been exploited before to the best of our knowledge. With a larger data set and more evenly distributed data points, we see the potential for an improved accuracy of this method.

With the availability of thermostable ancestral decarboxylases, the application of DES offers a potential 2-fold benefit: The solubility of the PAD substrates is higher in DES (Figure 2), and DES have been shown to exert a further stabilizing effect on proteins.^47^ The ancestral PAD N31 showed high activity in DES/water mixtures and maintained activity in the presence of 70% (v/v) DES/buffer and at 60 °C, while *Bs*PAD can perform decarboxylation in 50% (v/v) DES/buffer and at 30 °C. This allows applications in homogeneous solutions, which is beneficial, among others, for continuous flow setups.^4^ With N31, a 5-fold higher substrate loading could be achieved for the biocatalytic conversion of ferulic acid in a homogeneous monophasic solution, compared to the highest substrate loading reported so far.^4^ We did not investigate higher substrate loadings as the well-known product inhibition of PAD was expected to hinder full conversion; nevertheless, we are confident that a combination with in situ product removal will increase the volumetric yields further.^48^

The ability of the resurrected PAD N31 to perform decarboxylation reactions at higher temperatures and higher DES concentrations favors industrial applications. Simultaneously, the possibility of working with a clear solution instead of a slurry is advantageous for application in continuous flow systems.

**Figure 7:**
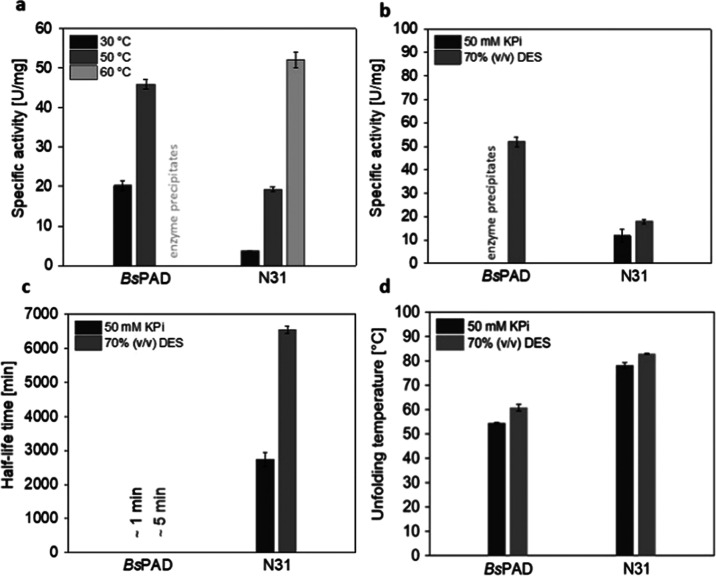
Activity, half-life time, and thermal stability of N31 and *Bs*PAD. (a) Specific activities of *Bs*PAD and N31 toward 10 mM ferulic acid were determined at 30, 50, and 60 °C in buffer (50 mM KPi, pH 6). (b) Specific activities of *Bs*PAD and N31 at 60 °C in buffer and 70% choline chloride/glycine (DES). (c) Half-life times of *Bs*PAD and N31 by incubating aliquots of the enzymes at 60 °C in buffer or DES and determining the residual activity at 30 °C. (d) Thermal stability determined by CD spectroscopy at 199 nm when in buffer and at 224 nm when in DES.

The specific activity of N31 increased notably from 30 to 60 °C, while *Bs*PAD immediately precipitated at 60 °C. Thus, N31 bears all of the features of a thermostable enzyme (Figure 7a). *Bs*PAD shows a specific activity of 20 U/mg at 30 °C and an initial rate of 45 U/mg at 50 °C. We note, however, that the low stability of *Bs*PAD at 50 °C precludes application at this temperature. In contrast, N31 shows a specific activity of 50 U/mg at 60 °C, a temperature where the half-life time exceeds days (Figure 7b). Therefore, engineering achieved a 2-fold increase in the specific activity for process applications. The superior stability of N31 compared to *Bs*PAD in both buffer and DES/buffer mixture is illustrated by the half-life time profile obtained at 60 °C (Figure 7c). N31 retained its stability for more than 3 days, while under the same conditions, *Bs*PAD rapidly lost its activity, preventing reliable half-life time determination.

Both enzymes retained their stability for a longer period when preincubated in a DES/buffer mixture, enhancing the hypothesis that DES components can stabilize enzymes. In addition to the half-life time, the thermal stability of the enzyme also increased in DES. Both N31 and *Bs*PAD exhibited an ∼6 °C increased unfolding temperature in 70% (v/v) DES/buffer as determined by circular dichroism spectroscopy (Figure 7d). To the best of our knowledge, unfolding temperatures measured in such a high DES concentration using circular dichroism spectroscopy are reported for the first time. Wu et al. report that DES could enhance the stability of horseradish peroxidase, which catalyzes the H_2_O_2_-dependent oxidation of a wide variety of organic compounds.^47^

**Figure 8:**
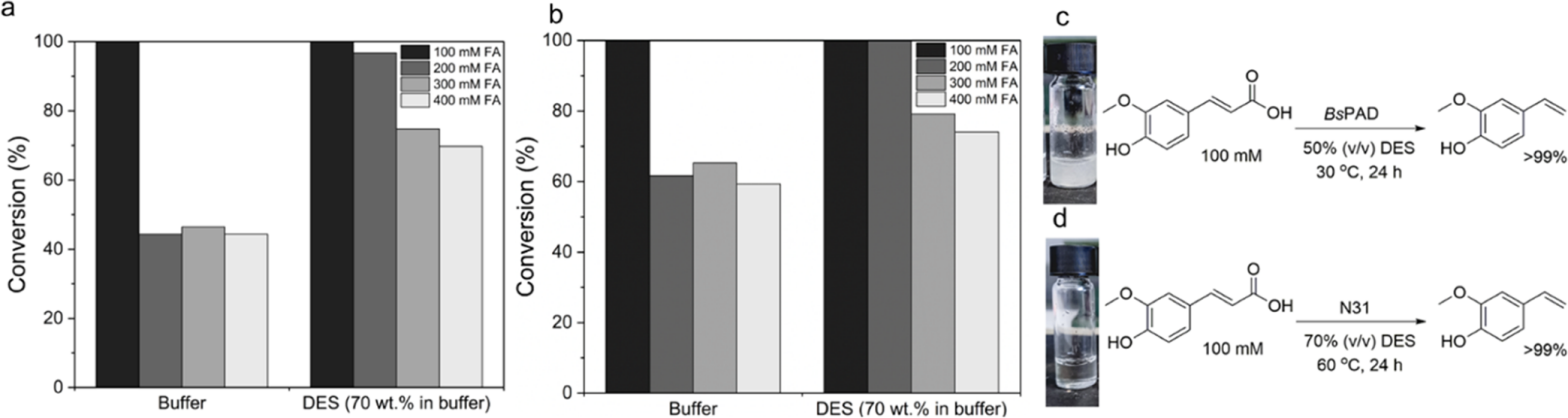
Decarboxylation of ferulic acid (a) with the extant *Bs*PAD in buffer and DES/buffer mixtures at 30 °C and (b) with the thermostable N31 in buffer and des/buffer mixtures at 60 °C. Reactions were initiated after the addition of 100 μL of CFE (100 mg/mL). The conversion was followed by HPLC. Comparison of the decarboxylation of ferulic acid with the extant *Bs*PAD (c) and thermostable N31 (d). The approaches are classified according to the starting solution provided for 100 mM ferulic acid (19.4 mg/mL) depending on the reaction conditions at which each enzyme displays optimum catalytic activity. Abbreviations: DES: 1 ChCl/2 Gly (mol/mol); buffer: 0.5 M KPi, pH 6.

In addition, DES can significantly increase the stability of an enzyme compared to conventional aqueous reaction systems.^49^ However, the effects of DESs on protein structure and activity have so far remained elusive. According to Hammond et al., DES/buffer mixtures of up to 30% (v/v) are still classified as ionic mixtures, taking full advantage of the DES properties regarding substrate solubility and enzyme stability.^50,51^ Applying temperatures of >60 °C to DES/buffer mixtures showed the best substrate solubility reported for homogeneous systems. While *Bs*PAD was inactive under these conditions, N31 showed a greatly enhanced half-life time. This aligns well with the observation that thermostable enzymes have more tolerance toward nonconventional solvents than mesophilic enzymes.^52^ For substrate loadings of 100 mM, quantitative conversions were achieved with both enzymes, with the difference that for N31, the starting solution was homogeneous, while for *Bs*PAD, it was a slurry (Figure 8a–d). Employing a higher buffer capacity not only enabled obtaining a higher conversion rate but also facilitated increased substrate solubility (compare Figures 2 and 8). *Bs*PAD is not active under the conditions used for N31 (Figure 8d), demonstrating the positive effect of the engineered variant. These results show that the buffer capacity influences both the enzyme productivity and the substrate solubility, which aligns well with the results of Pesci et al.^48^ Also, higher substrate loadings (200 mM ferulic acid) could be converted quantitatively with N31.

## Conclusions

The resurrection and expression of only ten randomly selected ancestral PAD sequences yielded significantly more thermostable enzymes than known extant decarboxylases. The overall topology and catalytic machinery are highly conserved. For the ancestors, structural attributes were calculated as a novel approach to train regression models to predict the unfolding temperatures of proteins. This method can be used to fit small data sets to regression models. In turn, the models can be used to identify proteins from the same family with a higher stability. One of the trained models was able to correctly identify 3 out of the 3 proteins with the highest unfolding temperature of the test data set. Therefore, these models show improved predictive power over global models that are not trained on specific data. This one-shot strategy is time-saving compared to rational design approaches, which depend on detailed knowledge of the structure and function of an enzyme. N31 is a phenolic acid decarboxylase with unparalleled thermostability, allowing it to work under conditions of higher temperature and thus much better substrate solubility, which is a critical factor for the space-time yield of a biocatalytic process. The high thermostability of N31 enables decarboxylation at reactions above 50 °C, where the viscosity of DES is lower. We note that the specific activity of N31 at 60 °C is twice as high as that of *Bs*PAD at 30 °C, which results in a much more active biocatalyst. As an example, decarboxylation was possible in a homogeneous, monophasic solution of a deep eutectic solvent, which is crucial for application in continuous setups. This highlights the superiority of the new artificial decarboxylase for future sustainable applications.

## Supporting Information Available

General experimental details, enzyme preparation, and expression yields; typical reaction procedures for activity and stability; α fold predictions and parameters; attributes calculated for each protein; catalytically important residues for PAD and variants; RMSF values computed by MD; and binding mode of ferulic acid in extant PAD and variants (PDF)

## Abbreviations

3D: 3-dimensional
ASR: ancestral sequence reconstruction
*Bc*PAD: phenolic acid decarboxylase from *Bacillus coagulans*
*Bs*PAD: phenolic acid decarboxylase from *Bacillus subtilis*
CaA: caffeic acid
CD: circular dichroism
ChCl: choline chloride
CuA: coumaric acid
DES: deep eutectic solvent
E. coli: *Escherichia coli*
FA: ferulic acid
Gly: glycerol
GRASP: graphical representation of ancestral sequence predictions
HPLC: high-performance liquid chromatography
JTT: Jones–Taylor–Thornton
MAE: mean absolute error
MD: molecular dynamics
MEGAX: molecular evolutionary genetics analysis X
ML: maximum likelihood
PAD: phenolic acid decarboxylase
PDB: Protein Data Bank
RMSD: root-mean-square deviation
RMSF: root-mean-square fluctuation
SA: sinapic acid
v/v: volume to volume
*Vn*PAD: phenolic acid decarboxylase from *Vibrio nigripulchritudo*
