# Supplementary material for: Stability Increase of Phenolic Acid Decarboxylase by a Combination of Protein and Solvent Engineering Unlocks Applications at Elevated Temperatures
Source: ACS Sustain Chem Eng. 2024 Feb 21;12(9):3575–84. doi: 10.1021/acssuschemeng.3c06513 (PMC10915792; doi:10.1021/acssuschemeng.3c06513)
Supplement: Supplementary file 1 — sc3c06513_si_001.pdf [file sc3c06513_si_001.pdf]

Supporting Information

Stability increase of phenolic acid decarboxylase by a combination of protein and solvent engineering unlocks applications at elevated temperatures

Kamela Myrtollari [a,b,c], Elia Calderini [a,†], Daniel Kracher [a,d], Tobias Schöngaßner [a], Stela Galušić [a], Anita Slavica [e], Andreas Taden [c], Daniel Mokos [f], Anna Schrüfer [f], Gregor Wirnsberger [f], Karl Gruber [d,f], Bastian Daniel [d,f,*], Robert Kourist [a,b,d,*]

[a]Institute of Molecular Biotechnology, Graz University of Technology, Petersgasse 14, 8010 Graz, Austria

[b]Austrian Centre of Industrial Biotechnology, ACIB GmbH; Petersgasse 14/1, 8010 Graz, Austria

[c]Adhesive Technologies, Henkel AG & Co. KGaA, Henkelstr. 67, 40191 Düsseldorf, Germany

[d]BioTechMed-Graz, Mozartgasse 12/II, 8010 Graz, Austria

[e]Faculty of Food Technology and Biotechnology, Department of Biochemical Engineering, University of Zagreb, Pierottijeva 6, HR-10000 Zagreb, Croatia

[f]Institute of Molecular Biosiences, University of Graz, NAWI Graz, Humboldtstraße 50/3, 8010 Graz, Austria

Corresponding authors: *Bastian Daniel (bastian.daniel@uni-graz.at), *Robert Kourist (kourist@tugraz.at)

Number of pages: 27, Number of figures: 13, Number of tables: 7

Table of Contents

Table of Contents 2
Experimental Procedures 3
Materials 3
Ancestral sequence reconstruction and phylogenetic analysis 3
Expression of *Bs*PAD and ancestral proteins 3
Preparation of Cell-Free Extract and Enzyme Purification 4
Gravity column purification of the six ancestors used for the test data set 4
Determination of the specific activity in buffer and DES/buffer mixtures 5
Determination of the half-life time 5
Circular Dichroism Spectroscopy 5
Determination of the experimental solubility 6
PAD-Catalyzed Decarboxylation of Ferulic Acid 6
Determination of activity using a spectroscopic assay 7
Preparation of DES 7
High-Performance Liquid Chromatography 7
Results and Discussion 8
AlphaFold predictions 15
Replica exchange molecular dynamics simulation 15
Salt bridge networks 15
Hydrogen bond networks 16
Hydrophobic cluster 16
Surface properties 16
Network analysis 16
Correlation and multiple linear regression 19
Code availability 19
Protein crystallization 20
Molecular Docking 22
Sequences 22
References 25

Experimental Procedures

Materials

Chemicals, cultivation media components, and reagents were purchased from MERCK (Darmstadt, Germany), VWR (Radnor, PE) and Enamine (Kyiv, Ukraine). Ferulic acid was used as received. The BCA protein quantification kit and reagents for molecular biology were purchased from Thermo Scientific (Waltham, MA). Choline chloride and glycerol were purchased from Sigma Aldrich. Protein and DNA concentrations were measured using a Nanodrop 2000 UV-Vis spectrophotometer (Thermo Scientific).

Ancestral sequence reconstruction and phylogenetic analysis

A set of 150 unique amino acid sequences encoding putative phenolic acid decarboxylases was collected from a homology search using BLAST[1], *Bs*PAD was used as query. After removing duplicate sequences, a multiple sequence alignment was constructed using Clustal Omega.[2] A few rounds of alignments were done taking care to remove sequences that would results in large gaps with a single sequence and sequences that did not have catalytically relevant residues. A phylogenetic tree was built with MEGA X[3] using the maximum likelihood method with 1000 bootstrap replicates. The ancestral sequences were inferred using the online version of GRASP, the MSA and phylogenetic tree were uploaded as inputs. The Jones-Taylor-Thornton (JTT) evolutionary rate model was used for the inference, as recommended by the GRASP authors, although other evolutionary models are available.[4,5]

Expression of *Bs*PAD and ancestral proteins

The synthetic genes corresponding to the ancestral enzymes were purchased from Twist Biosciences (South San Francisco USA). They were then transformed, checked for correct insertion by sequencing and expressed in *E. coli* BL21 (DE3) strains.[6] Transformants were inoculated in 5 mL LB medium containing 5 µg/mL kanamycin and grown at 37 °C and 130 rpm overnight. These cultures were used to inoculate TB-Kan medium (400 mL) where cells were grown at 37 °C and 130 rpm until OD_{600} of 0.5−0.7 was reached. To initiate expression 0.1 mM of IPTG was added followed by incubation at 20 °C and 120 rpm for 20−24 h. The cells were

harvested by centrifugation for 15 min, 4500 rpm at 4 °C. The resulting pellet was washed with 50 mM potassium phosphate buffer, pH 6 and stored at −20 °C.

Preparation of Cell-Free Extract and Enzyme Purification

To prepare the cell-free extracts, the pellet was resuspended (100 mg mL^{-1}) in 50 mM potassium phosphate buffer, pH 6 and the cells were disrupted by sonication (Branson Sonifier 250; 5 min, Duty Cycle 5, Output control 50%). After centrifugation for 20 min, 11 000 rpm at 4 °C, the supernatant was sterile filtered and used for the reaction. For freeze-drying, the obtained cell-free extract in the reaction buffer was shock-frozen in liquid nitrogen, and a vacuum was applied overnight (AdVantage Pro Lyophilizer, SP Scientific). For the purification of the enzyme, the pellet was resuspended in 20 mM Tris−HCl, 300 mM NaCl, 5 mM imidazole, pH 7.4. After a sonication and centrifugation step, the supernatant was sterile filtered and underwent His-tag purification following the manufacturer's instructions (Ni Sepharose 6 Fast Flow, GE Healthcare). All enzymes were purified by Ni-affinity chromatography using an ÄKTA pure protein purification system (chromatographic equipment from GE Healthcare Life Sciences, Austria). The buffer of the collected elution fractions was exchanged to 50 mM potassium phosphate buffer, pH 6 via over overnight dialysis. The protein concentration was determined with a BCA Protein Assay Kit (ThermoFisher Scientific) or by measuring absorbance in NANODROP at 280 nm, and the purified enzymes were stored in 10-20% glycerol at −80 °C. Protein purity was confirmed with SDS-PAGE.

Gravity column purification of the six ancestors used for the test data set

For purification the frozen pellets were resuspended in Buffer A (40 mM Tris−HCl, 300 mM NaCl, 30 mM imidazole, pH 7.4) and the cells lysed by sonication (Branson Sonifier 250; 5+2 min, Duty Cycle 5, Output control 50%). After centrifugation (30000g, 20 min, 4 °C) the clear cell extract was loaded on pre-equilibrated Ni-Sepharose columns and purified according to the manufacturer's manual (Ni Sepharose 6 Fast Flow, GE Healthcare). After elution the buffer was exchanged to 50 mM potassium phosphate buffer, pH 6 using PD-10 desalting columns (GE Healthcare). The concentration was then determined at 280 nm using Nanodrop, or with the

Bradford Protein Assay at 595 nm and the purity confirmed by SDS-Page. Finally, the purified enzymes were flash frozen and stored in 500 µl aliquots at – 80 °C.

Determination of the specific activity in buffer and DES/buffer mixtures

All reactions were performed on a 1 mL scale in 1.5 mL Eppendorf tubes at 50 and 60 ºC and in 2 mL 96-well plate at 30 ºC. C. 50 µL of ferulic acid (200 mM in DMSO, stock) were mixed with reaction buffer (50 mM KPi, pH 6) to reach a final concentration of 10 mM. The substrate solution was preincubated at the studied temperature before the respective amount of the purified enzyme (typically 2−10 µg) was added to achieve a final reaction volume of 1 mL. 150 µL of the sample were taken after defined timespans and were quenched by adding 150 µL acetonitrile containing 400 mM HCl. The supernatant was directly used for HPLC- UV measurements. Calibration was done for substrate decrease. Samples were performed in duplicates and analyzed by HPLC-DAD. When the reaction was performed in 70% (v/v) DES, 150 µL of each sample was diluted with 150 µL of ACN, followed by the addition of 200 µL reaction buffer to break the formed emulsion. Samples were thoroughly mixed and centrifuged, and the supernatants were used for activity measurements. One unit of enzyme activity was defined as 1 µmol product formation per minute (U) per mg of protein (U/mg).

Determination of the half-life time

The half-life time of the studied PADs was determined by incubating 1.0 µg/µL of purified PAD at 60 °C in >90% (v/v) DES (1 ChCl/ 2 Gly) or 100% buffer (50 mM KPi, pH 6). Samples of 200 µL were incubated for different time spans and cooled to room temperature for equilibration. The stability was measured in 1 mL reactions at 30 °C and 600 rpm (50 mM KPi pH 6, 10 µg PAD) by HPLC. More specifically, 150 µL of sample was taken from the reaction for the activity assay at 30 °C, as described above. Half-life times were determined from plots of the natural logs of residual activities versus the incubation time. Each reaction was performed in duplicates.

Circular Dichroism Spectroscopy

To determine the unfolding temperatures proteins were diluted in 50 mM potassium phosphate buffer, pH 6 to a final concentration of 0.1-0.4 mg/ml. Unfolding temperatures were determined

via a temperature interval scan measurement at a wavelength of 199 nm (*Bs*PAD, N0, N2, N4, N5, N31, N55, N80, N122, N124 and N134) or 224 nm (N10, N16, N39, N71, N100 and N146) with the protein dissolved in buffer or DES- buffer mixtures using a JASCO J-1500 CD spectrophotometer (JASCO Cooperation, Tokyo, Japan) equipped with a 1 mm path length quartz cuvette. Curve fitting was performed using the JASCO Spectra Manager 2.13.00. Spectra of *Bs*PAD in 50 mM phosphate buffer, pH 6, were collected in the far-UV range between 180 and 260 nm using 0.1 mg/mL of purified protein. Measurements carried out in the presence of DES (50% or 70% (v/v) in buffer) contained 0.3 mg/mL of enzyme and were collected between 200 and 260 nm. The temperature scan interval measurement in DES was recorded at 202 nm for *Bs*PAD and at 224 nm for N31. All spectra were recorded in triplicates and corrected for the buffer and DES contributions. The enzyme solutions were equilibrated at room temperature for 20 min and degassed in a vacuum chamber prior to all measurements.

Determination of the experimental solubility

The solubility of ferulic acid against buffer and DES-buffer mixtures was determined at 30 °C, 50 °C and 60 °C. For the measurement, an excess amount of ferulic acid was added to each medium (50 mM KPi pH 6, 50% or 70% v/v DES/buffer). The DES used for this study is ChCl/Gly (1/2 mol). Saturated samples were incubated at the respective temperature for 3 hours under constant stirring (600-700 rpm). Samples were cleared by centrifugation for 10 min at maximum speed (13.200 rpm) to remove undissolved substrates. 150 µL of diluted (1:10) samples were mixed with 150 µL acetonitrile and then centrifuged for 5 minutes. 200 µL were used for the HPLC analysis, which allowed quantification of the dissolved ferulic acid by comparison with a standard curve. All samples were measured in three independent measurements.

PAD-Catalyzed Decarboxylation of Ferulic Acid

Ferulic acid (10 mM) and lyophilized *Bs*PAD (6 mg) were added to 1000 µL solution containing 70% (v/v) of 1 ChCl/2 Gly (mol/mol) and 30% (v/v) 50 mM KPi, pH 6.0 in a 2.0 mL glass vial. The reaction was shaken at 250 rpm and 30 °C for 24 h. To monitor the reaction, aliquots of 150 mL were periodically removed and diluted with 150 µL acetonitrile (400 mM HCl). After centrifugation (5 minutes, 13.200 rpm), the concentration of residual ferulic acid was determined

by HPLC. All samples were measured in triplicates, and the activities were calculated from the initial slope/endpoint measurements.

Determination of activity using a spectroscopic assay

The activity of the six ancestors used for the test dataset and the extant PAD from *Vibrio nigripulchritudo* was determined using a microtiter plate assay based on the absorbance of the substrates.[7] For the assay 200 mM substrate stock solutions of ferulic acid, caffeic acid, coumaric acid and sinapic acid were freshly prepared in DMSO and further diluted in 50 mM potassium phosphate buffer, pH 6. Next, different substrate dilutions between 0.5 and 10 mM were prepared, and the absorbance measured between 230 nm and 550 nm. From this data the extinction coefficients at the respective wavelengths were calculated (Table S6). To measure the activity of the enzymes, 50 µl of enzyme solution was prepared in microtiter plates heated to 30 °C and 50 µl of preheated substrate solution was added to start the reaction. The plate was then immediately placed into the plate reader (FLUOstar Omega,BMG labtech, Offenburg Germany) and the decrease in absorbance followed every 20 sec over 10 min. For calculations the initial rate, containing at least 3 measurement points, was used. All measurements were carried out in technical triplicates and three different enzyme concentrations.

Preparation of DES

The ammonium salt, ChCl, and the hydrogen-bond donor, glycerol, were mixed in a molar ratio of 1:2 under constant stirring at 80 °C for 2 h until a colourless and transparent liquid was obtained. DES-containing aqueous solutions were prepared by dissolving DES in 50 mM KPi, pH 6.0 to reach final concentrations of 50 or 70% (v/v).

High-Performance Liquid Chromatography

All HPLC analyses were performed on an Agilent Technologies 1100 Series with an autosampler, and DAD detector connected to a reversed-phase Nucleodur C18 Pyramid column (5 µm, 250 × 4.6 mm, Macherey Nagel). A mixture of acetonitrile and double-distilled H_2O was used as eluent (40% (v/v) acetonitrile, isocratic, 1 mL min^{-1}. Absorption was detected simultaneously at 305 nm. For the calibration, a 10 mM stock of substrate in 50 mM KPi, pH 6 was used to prepare dilutions

in the range of 1−10 mM using the same buffer. The resulting standard curves are shown in Figure S13. For measurements, 150 µL of each sample was diluted with 150 µL acetonitrile containing 400 mM HCl and after a centrifugation step (5 minutes, 13.000 rpm, T_{room}) 200 µL of the supernatant was used for the analysis. When measuring in aqueous DES solutions, 150 µL of each sample was diluted with 150 µL acetonitrile and 200 µL buffer to break the formed emulsion. All calibration samples were prepared in triplicates.

Results and Discussion

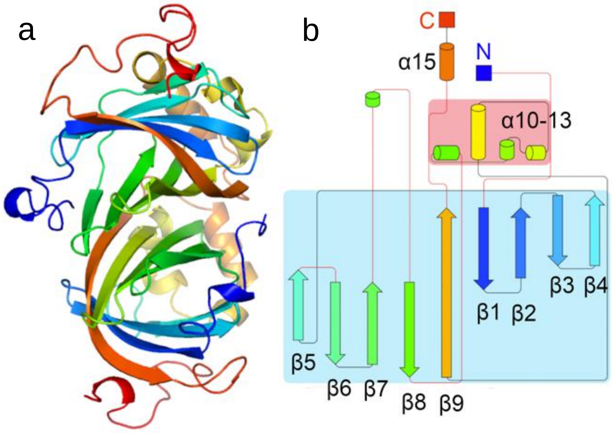

Figure S1. PAD-dimer and topology plot colored from blue to red from N- to C-terminus. The core of the monomers is formed by two mutually perpendicular beta-sheets with the strand order β9-β1-β2-β3-β4 and β5-β6-β7-β8-β9 with β9 belonging to both sheets as indicated in B. The latter sheet forms the interface of the PAD homodimer from two monomers A and B that are related by an improper twofold axis with residues from β5A interacting with β9B, β6A with β8B, and the central strands β7A with β7B and vice versa.

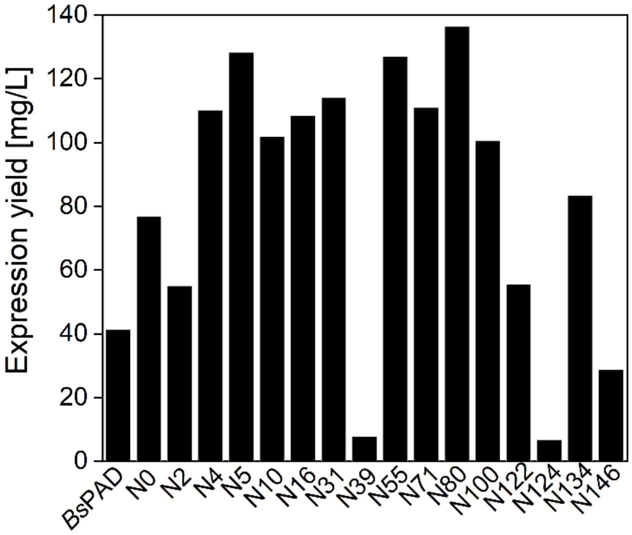

Figure S2. Expression yield of the purified extant *Bs*PAD and ancestral PADs.

Table S1. Origin of the extant enzymes of N31.

Accession	Gene ID	Organism	Environment	Reference
>WP_130611702.1	1583031843	Muricauda hymeniacidonis	Sponge/Cheongpo Beach	8
>WP_137939079.1	SCOH00000000	Chitinivorax sp. B	Alpine spring water	9
>WP_107545948.1	1378600299	Staphylococcus xylosus	Human skin	10
>WP_004401177.1	490536014	Vibrio nigripulchritudo	Schrimp pond	11

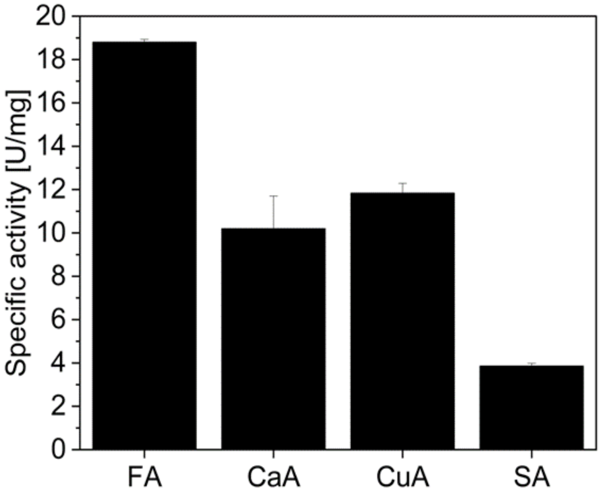

Figure S3. Specific activity of phenolic acid decarboxylase from *Vibrio nigripulchritudo* measured with a spectroscopic assay.

Table S2. Unfolding temperature of N31 and single-cite variants prepared with PoPMuSiC.

PAD	T_m (ºC)
N31	78
Glu28Tyr	52
Asp38Tyr	55
Lys67Ile	71
Asp77Phe	38
Gly91Phe	67

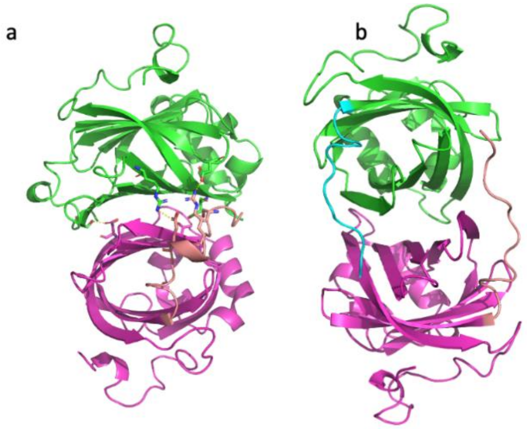

Figure S4. Contribution of the N-terminal loop to the dimerization. N55 dimer with one monomer in green and one in pink (a). The N-terminal loop of the pink dimer is indicated in salmon, the polar contacts as yellow dashed lines. The N134 monomers are depicted in green and pink, with a cyan and salmon colored N-terminal loop, respectively (b). While the dimer interface is mainly formed by the beta sheets, in N134, the 35 residues long N-terminal loop crosses to the respective counterpart in the PAD dimer. The residues 1 to 24 cannot be resolved, probably due to the unstructured nature of this loop. Similarly, N55 has a 35 residues long N-terminal loop that can be resolved in the electron density starting from residue 24, which contributes two salt bridges and one polar contact per loop to the dimerization.

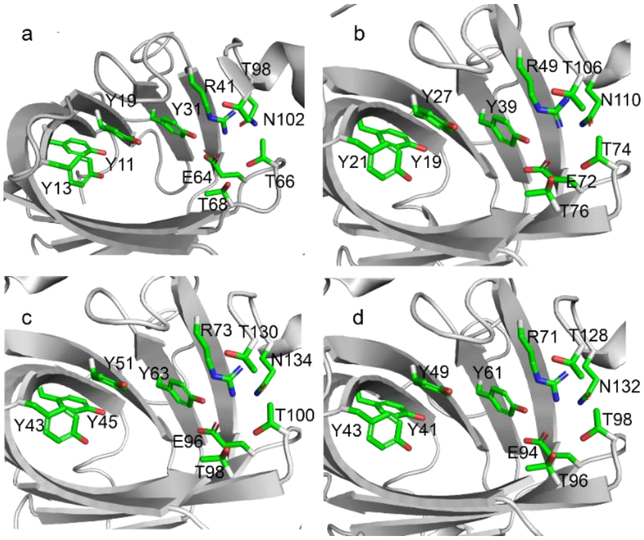

Figure S5. Catalytically important residues in the PAD variants *Bs*PAD (a), N31 (b), N55 (c), and N134 (d). All structures are visualized using PyMOL.

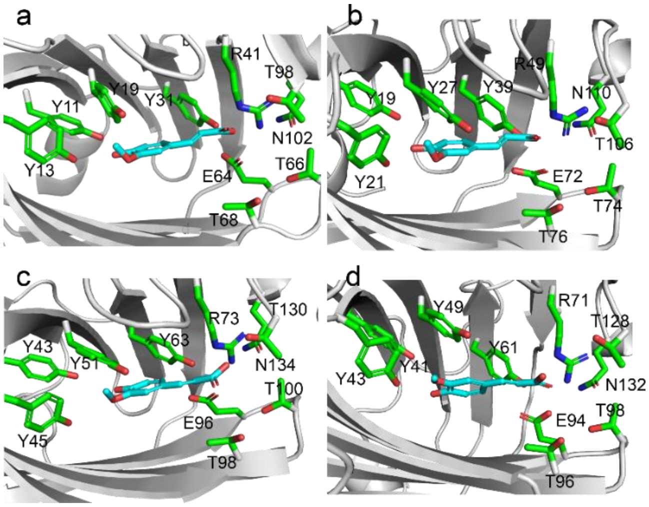

Figure S6. Putative binding mode of ferulic acid (cyan) in *Bs*PAD (a), N31 (b), N55 (c) and N134 (d). All structures are visualized using PyMOL.

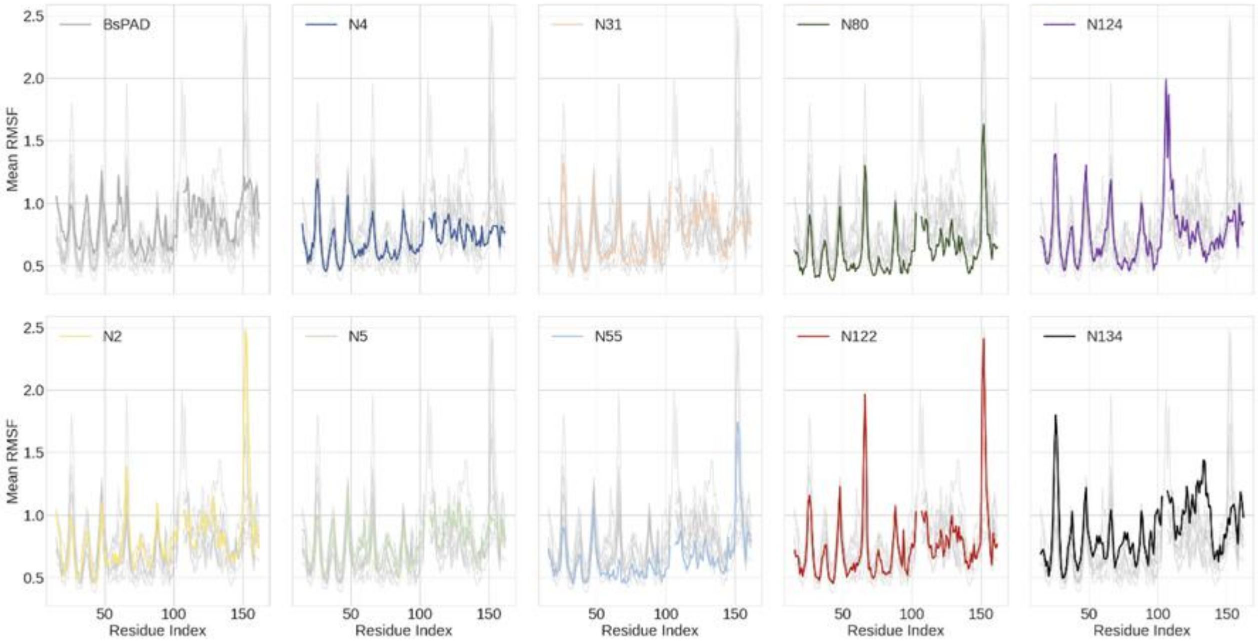

Figure S7. C-alpha RMSF values of each residue in a single chain mapped on a multiple sequence alignment. In each panel, the RMSF-values obtained for one protein are highlighted, while the curves of all other proteins are shown in grey. The different ancestorial proteins are named according to their node numbers. The first and the last 25 residues were omitted because they are not comparable due to their greatly increased flexibility and would overpower the more meaningful values of the cores of the proteins in the analysis.

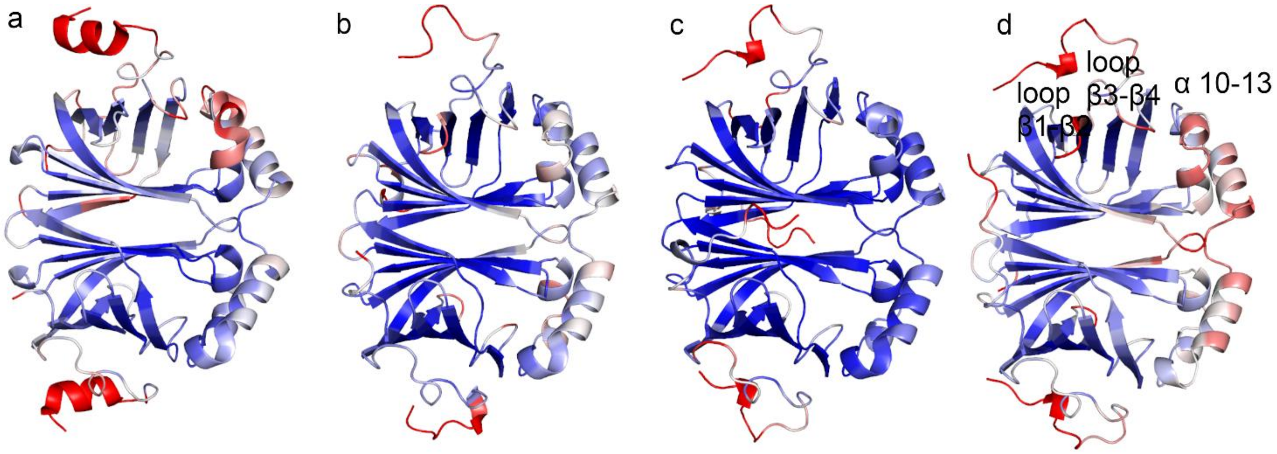

Figure S8. PAD dimer coloured according to the RMSF computed by MD. *Bs*PAD (a), N31 (b), N55 (c), and N134 (d). Rigid regions are coloured blue, flexible regions red. All structures are visualized using PyMOL.

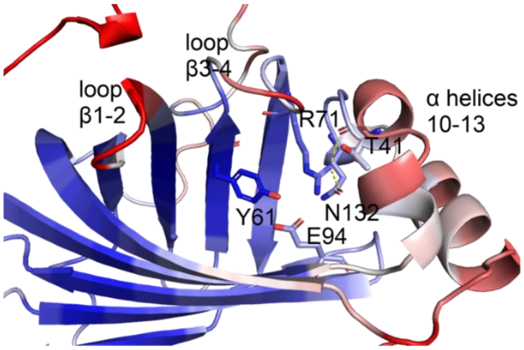

Figure S9. Close up on the regions with elevated flexibility of N134 that are putatively involved in opening and closing the active site (loop from beta-strand 1 to beta-strand 2 and loop from beta-strand 3 to beta-strand 4, and alpha-helices 10-13 that harbour second shell residues that are involved in the positioning of the catalytic active Arg. The structure is visualized using PyMOL.

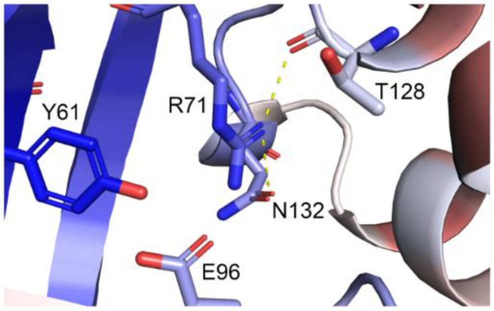

Figure S10. Interaction of Thr128 and N132 from alpha-helices 10-13 with catalytic active Arg71 from N134. The structure is visualized using PyMOL.

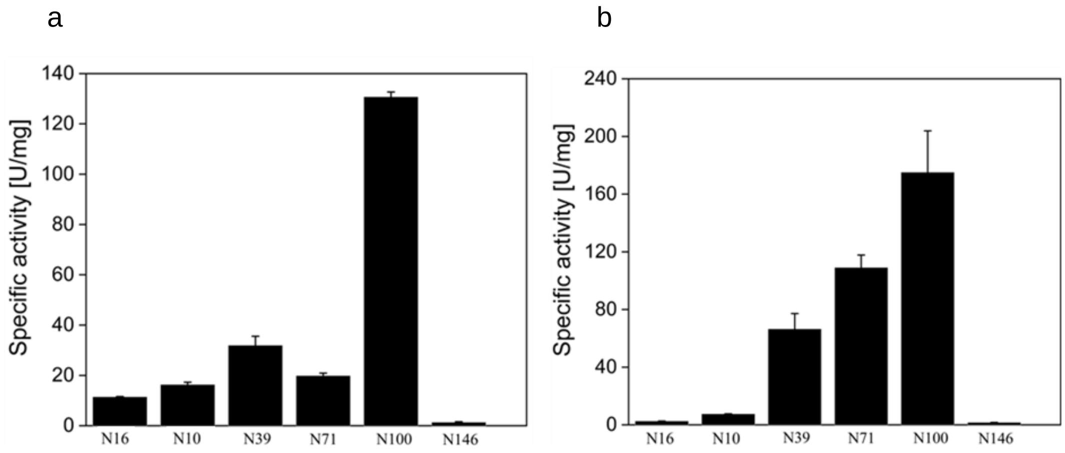

Figure S11. Specific activity of the new set of the PAD-ancestors towards 1 mM (a) and 5mM (b) of ferulic acid measured with a spectroscopic assay.

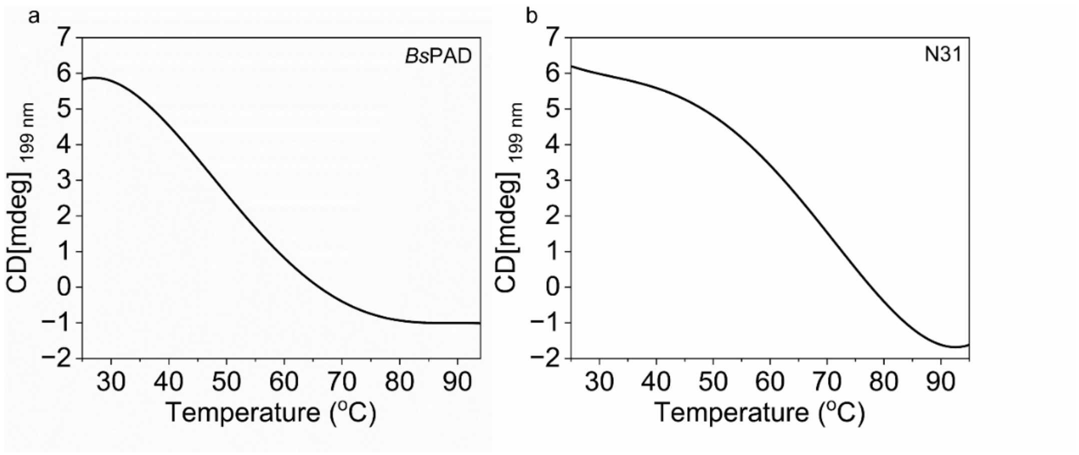

Figure S12. Fitted CD-melting of *Bs*PAD (a) and N31 (b) measured at 199 nm. Final enzyme concentration: 0.1 mg/mL. CD traces were measured within a range of 25°C to 95°C.

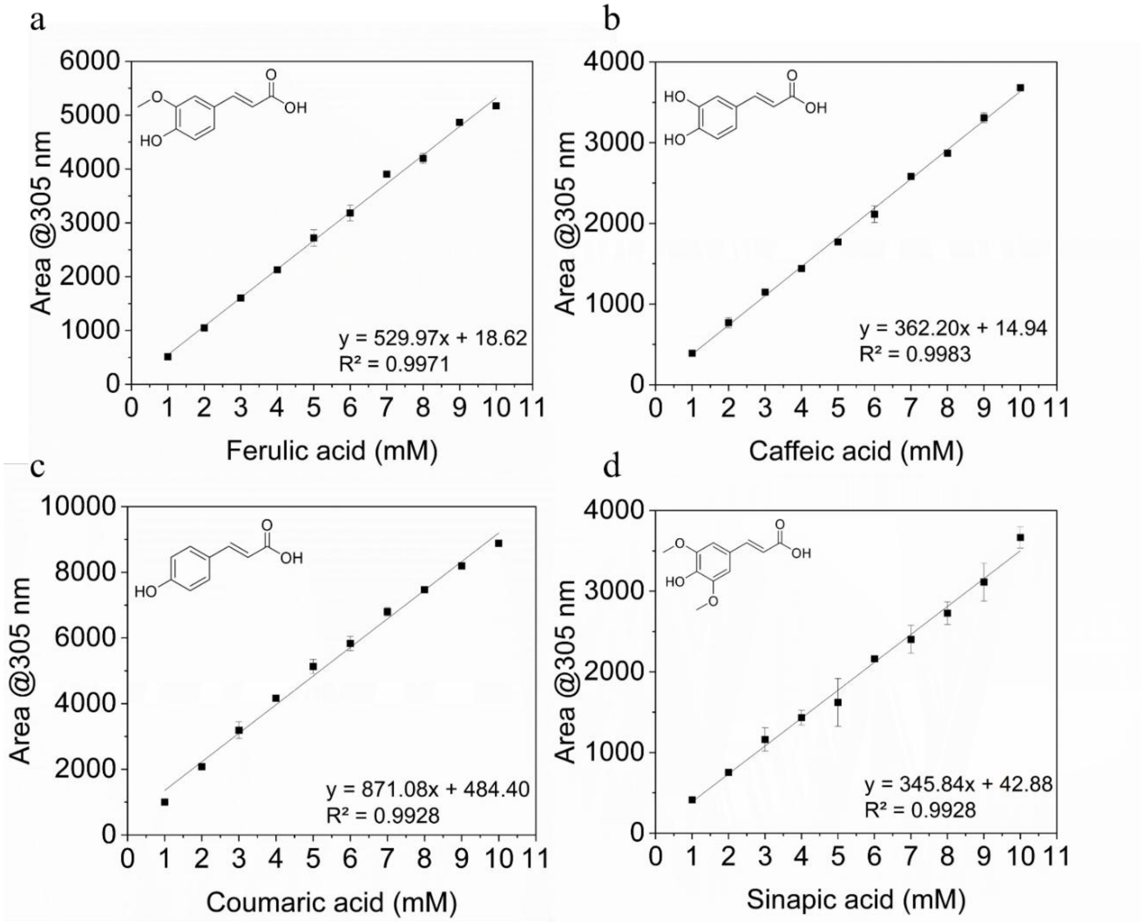

Figure S13. Calibration curves for ferulic acid (a), caffeic acid (b), coumaric acid (c), and sinapic acid (d) determined via HPLC. Injection volume: 2 µL, $\lambda_{detection}$ = 305 nm.

AlphaFold predictions

Structures of the proteins for which no crystal structure was available (N2, N4, N5, N80, N122, N124) were predicted using AlphaFold[12], with the model preset set to multimer and a max template date of 2022-05-01. The input was two times the same amino acid sequence to generate dimers since the available crystal structures all show a dimeric quaternary structure.

Replica exchange molecular dynamics simulation

The replica exchange molecular dynamics simulations were carried out using Schrödinger's Desmond replica exchange algorithm.[13] The systems were prepared by adding SPC water as solvent as well as Na+ ions to neutralize the charge. The systems were then minimized by using the OPLS4 force field. These prepared systems were used to perform replica exchange molecular dynamics simulation with 10 replicas per structure in a temperature range from 300–310 K. A parallel tempering model (REMD) with a linear temperature profile was used. The simulations were carried out over 10 ns with a recording interval of 10 ps which results in a total of 1000 frames per protein. As an ensemble class NPT was used with a pressure of 1.01325 bar. To calculate nonbonded forces, a RESPA integrator with a time step of 2 fs was used. The short- range forces were updated every time step and the long- range forces every 3-time steps. As a short-range cut-off, 9 Å was used. Periodic boundary conditions were used.

Root mean square fluctuations (RMSF) were derived from Schrödinger's maestro simulation interaction diagram and refer to the root mean square fluctuations of the C-alpha atoms in the protein main chains. The RMSF values per residues' C-alpha were averaged over the 10 replicas to yield one RMSF value per residue.

Salt bridge networks

Salt bridges are one of the stabilizing interactions in proteins. These emerge when a negatively and a positively charged side chain atom inside a protein are closer than 3.5 Å. The side chains in question belong to arginine, lysine, histidine, aspartate, and glutamate. To calculate the salt bridge networks, we compute the distances between all negatively charged side chain atoms to all positively charged side chain atoms. When they are closer or as close as the cut-off, they get

marked as interacting. Subsequently, pairs with at least one common residue are grouped into networks.

Hydrogen bond networks

To calculate the hydrogen bond networks, the distance between the hydrogen atom of the donor and the electronegative acceptor atom was calculated. This distance must not exceed 2.5 Å. Furthermore, the angle between donor, donor hydrogen, and acceptor was calculated. This angle must not be less than 120°.[14] Residues that fulfil these requirements are seen as H-bond forming and clustered in the same way as described for salt bridges.

Hydrophobic cluster

To find the hydrophobic clusters that play a key role in terms of protein stability[15], we calculate the distances between the side chain atoms of the amino acids leucine, isoleucine, and valine. If the distance between two side chain atoms is less than 6.56 Å, this pair is counted as a hydrophobic interaction. The pairs are then clustered in the same way as for the salt bridges and hydrogen bonds. The hydrophobic cluster area is calculated by summing up the surface accessible side chain area of each residue in a cluster.

Surface properties

Several sources show that modifying the protein surface can lead to increased protein stability.[16,17] In order to find residues that are located on the protein surface, a solvent shell is built around the protein and residues that can access this shell are searched for. A number of different attributes are then calculated for this group of residues (Table S3).

Network analysis

In order to obtain better comparable structures, we predicted AlphaFold 2 models of all enzymes and did not use the available crystal structures for this analysis. To calculate all attributes with structures from the same origin, ColabFold[18] was used to predict the dimeric structures of all proteins with its default settings and amber relax. Using these structures, the different attributes (salt bridges, hydrogen bonds, hydrophobic cluster and surface properties) of the proteins were

calculated (Table S4) and ranked according to the correlation of the data to the stability (Table S3). Additionally, the pipeline creates scripts that are executable in PyMOL, to select and present the respective residues that contribute to a given structural attribute as sticks (compare Figure 5 panels b, c and d). As the PADs form homodimers, every residue contributing to an attribute should be identified in both monomers and due to the nature of the dimerization (compare Figure S1) the arrangement of the attributes should by symmetrical. This was verified using PyMOL by visual inspection of the respective structures of the ancestors after applying the pipeline-generated scripts to them (compare Figure 5). If a symmetrical arrangement of the respective attributes can be calculated using the pipeline, we anticipate, that model quality is sufficient, and the code is functional to quantify the respective attributes for further calculations.

Table S3. The best three properties of the respective network analyses (hydrogen bonds, hydrophobic clusters, salt bridges, and surface properties) with the highest correlation to the experimentally determined unfolding temperatures of the test dataset when using AlphaFold models as basis to calculate the attribute values. All attributes are shown in Table S4.

**Network**	**Data**	**Pearson's R**	**p Value**
H-bond	MAX NWS HB	0.3756	0.2290
H-bond	SUM BPN HB	-0.3407	0.2785
H-bond	MEAN BPN HB	-0.2949	0.3521
Hydrophobic cluster	MAX CA	0.6212	0.0311
Hydrophobic cluster	MAX CC	0.5982	0.0399
Hydrophobic cluster	SUM CC	0.5761	0.0499
Salt bridges	SUM NWS SB	-0.3604	0.2498
Salt bridges	SUM IA SB	-0.3438	0.2739
Salt bridges	MAX NWS SB	-0.2956	0.3509
Surface properties	SUM HYDROPATHY	0.6313	0.0277
Surface properties	POS CHARGED	-0.5962	0.0408
Surface properties	NEG HYDROPATHY	0.5163	0.0857

Table S4. Network attributes deduced from the structures. Of these, the maximum (MAX) number and the mean (MEAN) number of all clusters were determined. Furthermore, the sum (SUM) over all clusters and networks of the attributes was calculated. Surface attributes always represent the sum of their appearance.

**Attribute**	**Description**
CA	Sum of the surface accessible sidechain areas of each residue in cluster
CC	Hydrophobic contacts inside a cluster
BPN HB	Number of hydrogen bonds inside a H-bond network
NWS HB	Number of residues inside a H-bond network
IA SB	Number of salt bridges inside a network
NWS SB	Number of residues forming salt bridges inside a network
ICH, ICB, ICSB	Inter-chain hydrophobic interactions, hydrogen bonds, or salt bridges
CHARGED	Positive (pos) or negative (neg) charged surface residues
CHARGED RES	Charged surface residues
HYDROPATHY	Positive (pos), negative (neg) hydropathy surface residues
SF AREA	Surface accessible side chain area surface residues

Table S5. Melting temperatures (Tm, in °C) of wild-type and ancestral proteins determined with circular dichroism (CD) spectroscopy and calculated using the prediction tools SCooP and DeepSTABp.

	CD	SCooP	DeepSTABp
*Bs*PAD	54.5	67.5	48.6
*Vn*PAD	57.7	64.3	47.9
N0	55.6	62.9	47.8
N2	56.1	60.0	47.2
N4	55.2	63.6	49.0
N5	46.4	65.7	48.9
N10	57.3	66.2	48.7
N16	61.0	63.7	48.6
N31	78.1	64.6	48.1
N39	49.9	63.2	47.1
N55	57.8	64.4	47.8
N71	67.3	64.8	48.7
N80	65.4	65.3	47.8
N100	60.2	64.4	46.4
N122	60.9	62.3	48.2
N124	55.3	60.7	49.0
N134	72.7	58.8	50.0

N146	66.2	62.6	44.2

Table S6. Determination of the extinction coefficients used in the spectroscopic assay.

Compound	Wavelength (nm)	e [mM-1*cm-1]	SD (+/-)
1 mM Sinapic Acid	332 nm	6809.04	422.45
1 mM Caffeic Acid	328 nm	8195.07	223.47
1 mM Coumaric Acid	318 nm	7617.59	228.28
1 mM Ferulic Acid	330 nm	7063.77	368.52
5 mM Ferulic Acid	344 nm	1532.17	119.40

Correlation and multiple linear regression

To determine, which network attributes (Table S4) should subsequently be used as prediction basis, all 4-fold combinations of all attributes were used and the mean absolute error (MAE) of the predictions was calculated. As the attributes have a wide range of values, they are all scaled by subtracting their mean and dividing by their standard deviation. The predictions were done using a linear regression model, a random forest model with 10 decision trees where we chose a maximum depth of the trees of 3 and a k-nearest neighbours' regression, where 4 neighbours and their distance as weights were used to make predictions. To assess a model's performance with only 12 data points, a leave-one-out analysis[19] was performed where the linear regression is fitted to 11 of the 12 data points, and the 12th data point is used as the test set. This was done until each point was used once as a test. Errors for the test data were averaged, yielding the MAE. The MAE was used as a determining factor in choosing the best models. The leave-one-out analysis was done using scikit-learn.[20] The trained models with the lowest MAE were then fitted to the full dataset of 12 proteins. A set of six randomly selected ancestors, not used for model finding and training, was used as a test set to evaluate the performance of the models on unseen proteins. In order to compare the performance of our prediction models, we used SCooP[21] to predict the unfolding temperature of the test set proteins. Here we used their available online server but omitted the input of the host organism since it was unknown.

Code availability

The code to perform the same kind of analysis for different proteins as well as the scripts and data used to perform our analysis can be found on GitHub (https://github.com/ugSUBMARINE/structural-properties). Furthermore, an easy-to-use command line interface is provided.

Protein crystallization

Protein crystallization was conducted by vapor diffusion technique using Swissci plates (Swissci AG, Neuheim, Switzerland) and an Oryx8 robot (Douglas Instruments Berkshire, UK) employing the Index screen (Hampton Research, Viejo, USA). The proteins were prepared in 10 mM MES, 150 mM NaCl, pH = 6 buffer for crystallization. For N31, crystals were grown by mixing 0.5 µL lithium sulphate (0.2 M), BisTRIS (0.1 M, pH 5.5) and 25 % PEG 3350 with 0.5 µL N31 (10.00 mg/mL) on a Swissci triple well plates using the sitting drop method with a reservoir volume of 30 µL. Crystals appeared within 1-3 weeks and were frozen in liquid nitrogen without cryo protection for diffraction experiments. A data set with a resolution of 2.7 Å was collected in house that at a dual port system made up by a MetalJet X-ray source (Excillium, Kista, Sweden), a Venture D8 X-ray diffractometer (Bruker, Billerica, USA) and a Photon III detector (Bruker, Billerica, USA) at 100 °K. Data processing was performed with the Proteum3 program package (Bruker, Billerica, USA). For N134, crystals were grown by mixing 0.5 µL lithium sulphate (0.2 M), HEPES (0.1 M, pH 7.5) and 25 % PEG 3350 with 0.5 µL N134 (10.00 mg/mL) on a Swissci triple well plate using the sitting drop method with a reservoir volume of 30 µL. Crystals appeared within 1-3 weeks and were frozen in liquid nitrogen without cryo protection for diffraction experiments. A data set with a resolution of 2.67 Å was collected at our Bruker D8 Venture diffractometer at 100 °K as described above. For N55, crystals were grown by mixing 0.5 µL HEPES (0.1 M, pH 7.5) and ammonium sulfate (2.0 M) with 0.5 µL N55 (10.00 mg/mL) on a Swissci triple well plate using the sitting drop method with a reservoir volume of 30 µL. Crystals appeared within 1-3 days and were frozen in liquid nitrogen without cryo protection for diffraction experiments. A data set with a resolution of 1.6 Å was collected at the ID23-2 beamline of the ESRF in Grenoble (France) at 100 °K. Data processing was performed with the XDS program package. Additionally, data set for N55 of the same resolution was collected at the Bruker D8 Venture diffractometer at 100 °K as described above. Unit cell parameters and assigned space groups as well as data statistics are shown in Table S7.

Table S7. Data collection and refinement statistics. Statistics for the highest-resolution shell are shown in parentheses.

	**N55 (synchrotron)**	**N55 (home source)**	**N134**	**N31**
PDB code	8ADX	8C66	8A85	8B30
Resolution range (Å)	37.53 - 1.6 (1.657 - 1.6)	37.89 - 1.6 (1.657 - 1.6)	35.95 - 2.67 (2.765 - 2.67)	33.31 - 2.7 (2.797 - 2.7)
Space group	P 1 21 1	P 1 21 1	P 21 21 21	P 21 21 21
Unit cell (Å, °)	57.737 104.847 81.495, 90 109.865 90	57.9992 105.274 81.3174 90 109.77 90	97.3209 106.679 114.752 90 90 90	43.0694 59.9714 105.1 90 90 90
Total reflections	405300 (42883)	1153552 (75456)	489402 (41371)	53058 (5136)
Unique reflections	116564 (11733)	119180 (10814)	34571 (3380)	7901 (754)
Multiplicity	3.5 (3.7)	9.7 (7.0)	14.2 (12.2)	6.7 (6.8)
Completeness (%)	97.12 (98.32)	98.22 (88.97)	99.86 (99.94)	99.82 (100.00)
Mean I/sigma(I)	11.03 (2.91)	9.42 (2.18)	13.09 (2.70)	5.69 (1.79)
Wilson B-factor (Å^2)	17.57	12.70	36.50	47.46
R-merge	0.07811 (0.3753)	0.1584 (0.9154)	0.1672 (0.8962)	0.1435 (0.9337)
R-meas	0.09293 (0.44)	0.1677 (0.9864)	0.1734 (0.9347)	0.1555 (1.01)
R-pim	0.04972 (0.2277)	0.05381 (0.3557)	0.04549 (0.2625)	0.05802 (0.3743)
CC1/2	0.989 (0.862)	0.993 (0.613)	0.997 (0.868)	0.995 (0.683)
CC*	0.997 (0.962)	0.998 (0.872)	0.999 (0.964)	0.999 (0.901)
Reflections used in refinement	116496 (11730)	118658 (10728)	34553 (3380)	7896 (754)
Reflections used for R-free	5823 (581)	5946 (514)	1774 (169)	384 (43)
R-work	0.1958 (0.2109)	0.2334 (0.2719)	0.1932 (0.2735)	0.2139 (0.2759)
R-free	0.2386 (0.2836)	0.2721 (0.3319)	0.2320 (0.3545)	0.2751 (0.4194)
CC (work)	0.926 (0.837)	0.823 (0.291)	0.955 (0.862)	0.932 (0.807)
CC (free)	0.914 (0.713)	0.822 (0.248)	0.930 (0.766)	0.863 (0.420)
Number of non-hydrogen atoms overall	6187	6165	8289	2624
Number of non-hydrogen atoms of macromolecules	5452	5468	8013	2603

Number of non-hydrogen atoms of ligands	5	25	0	0
Solvent	730	672	276	21
Protein residues	674	676	998	317
RMS (bonds) (Å)	0.015	0.021	0.014	0.017
RMS (angles) (°)	1.85	2.22	1.80	2.47
Ramachandran favored (%)	98.35	98.50	97.36	97.12
Ramachandran allowed (%)	1.65	1.50	2.54	2.88
Ramachandran outliers (%)	0.00	0.00	0.10	0.00
Rotamer outliers (%)	0.35	1.38	2.62	12.63
Clashscore	2.44	3.83	1.92	10.66
Average B-factor ($Å^2$)	21.02	13.31	37.30	51.06
B-factor of macromolecules ($Å^2$)	19.22	10.59	37.59	51.21
B-factor of ligands ($Å^2$)	56.72	44.84	28.87	32.71

Data were analyzed with Aimless[22–24] in CCP4 Cloud.[25] The solvent content was estimated based on the calculated Matthews coefficient.[26] Phasing was performed using chain A of 4UU3 from the PDB as template for phaser MR.[27] Refinement was conducted by repetitive rounds of REFMAC[28] in the CCP4 cloud program package and manual model building in COOT.[29] Refinement cycles have also been performed with phenix.refine to improve the model quality. Models have been deposited with the PDB codes found in Table S7.

Molecular Docking
Molecular docking was performed using DiffDock with the default parameters.[30]

Sequences
Mature amino acid sequences given in black with N-terminal HIS-tag in red.

*Bs*PAD

MGSSHHHHHHSSGLVPRGSHMENFIGSHMIYTYENGWEYEIYIKNDHTIDYRIHSGMVA
GRWVRDQEVNIVKLTEGVYKVSWTEPTGTDVSLNFMPNEKRMHGIIFFPKWVHEHPEIT
VCYQNDHIDLMKESREKYETYPKYVVPEFAEITFLKNEGVDNEEVISKAPYEGMTDDIR
AGRL

*Vn*PAD

MGSSHHHHHHSSGLVPRGSHMRFDREDLSQFVGKSFIYNYDKGWRYELYVKNRTTIDY
RVHSGIVAGRWVKDQTVCLARVGENLYRVSWTEPTGTDVSLTLNLHDFVVHGAIYFPR
WIVNNPEKIACYQNEHLEEMEALRDAGPIYPTEIIDSFATLIYMRDCGPDNEQVIACPPSE
LPENYPFCLPDKNLLPESAAIA

N0

MGSSHHHHHHSSGLVPRGSHMTTASSPTPTQDLSGIVGKHLIYTYANGWQYEMYVKNE
NTIDYRIHSGMVGGRWVRDQQVHIVRLGDDVYKVSWTEPTGTSVSLAVDLAERRLHG
VIFFPQWVAQHPERTVCFQNDHLDEMRAYRDEGPTYPKLVIDEFATITFLEDCGPDDET
VIDCAPAELPAGYADRTN

N2

MGSSHHHHHHSSGLVPRGSHMTTASSSTHTEDLTGFVGKHLIYTYDNGWQYEMYVKN
ENTIDYRIHSGMVGGRWVRDQQVHIVRLGDDVYKISWDEPTGTTVSLAVNLAERRLHG
VIFFPQWIAQDPEKTVCFQNDHLDEMRAYRDAGPTYPKLVIDEFATITFMEDCGADDET
VIDCAPSELPAGYADRRN

N4

MGSSHHHHHHSSGLVPRGSHMSTHKEDLTGFVGKHFIYTYDNGWQYEIYVKNENTIDY
RIHSGMVGGRWVKDQQVHIVRLADDVYKISWTEPTGTDVSLTVNLAERKLHGTIFFPR
WIAEDPEKTVCYQNDHLPLMRAYRDAGPTYPKLVIDEFATITFMRDCGANNETVINCAP
SELPAGYADRRNGKSTLPNYPRIAK

N5

MGSSHHHHHHSSGLVPRGSHMMMQMKTLTDFIGTHFIYTYDNGWEYEIYVKNENTIDY
RIHSGMVGGRWVKDQKVHIVRLTDGVYKISWTEPTGTDVSLNFMLNEKKMHGVIFFPK
WVNEDPEKTVCYQNDHLPLMKEYREAGPTYPKLVVSEFATITFMRNCGANNETVINQA
PYPGMTADIRSGKSTFSNYKRINK

N10

MGSSHHHHHHSSGLVPRGSHMQMKTLKDFIGTHFIYTYDNGWEYEMYVKNEHTIDYRI
HSGMVGGRWVKDQEVDIVKLTEGVYKISWTEPTGTDVSLNFMPNEKKMHGVIFFPKW
VHEHPEITVCYQNDFIDLMEESREKYETYPKYVVPEFAKITYIGNAGVNNEKVIAQAPYE
GMTDDIRSGKLTFSNYKRINK

N16

MGSSHHHHHHSSGLVPRGSHMTKQFKTLDDFLGTHFIYTYDNGWEYEWYAKNDHTVD
YRIHGGMVAGRWVKDQEAHIVMLTEGIYKITWTEPTGTDVALDFMPNEGKLHGTIFFP
KWVQEHPEITVCYQNEHIDLMEESREKYETYPKLVVPEFATITYMGDAGQDNEDVISEA
PYEGMTDDIRNGKYFDENYKRINK

N31

MGSSHHHHHHSSGLVPRGSHMMSFDKEDLSGFVGKHFIYTYDNGWRYEIYVKNENTID
YRIHSGIVGGRWVKDQQVYIVRVADDVYKISWTEPTGTDVSLTVNLADYILHGTIFFPR
WIIENPEKTVCYQNDHLPLMRAYRDAGPTYPKEVIDEFATITFMRDCGENNETVINCPPS
ELPADYPDCLKGKT

N39

MGSSHHHHHHSSGLVPRGSHMSTFDKHDLSGFVGKHLVYTYDNGWNYEIYVKNDNT
MDYRIHSGIVGNRWVKDQRVYIVRVGESIYKISWTEPTGTDVSLIVNLGDKLFHGTIFFP
RWIINNPEKTVCFQNDHIPEMEAYRDAGPAYPTEVIDEFATITFVRDCGENNDSVINCAA
SELPADFPNNLK

N55

MGSSHHHHHHSSGLVPRGSHMSDAFESTRPEELTGFVGKHLIYTYDNGWQYEMYVKN
ERTIDYRIHSGMVGGRWVRDQLVHIVRLSDDVYKISWDEPTGTTVSVAVNLAERRLHG
VIFFPQWIAQDPKKTVCFQNDHLDEMRAYRDAGPTYPKLVIDEFATITFMEDCGADDET
VIACAPSELPAGYAARRN

N71

MGSSHHHHHHSSGLVPRGSHMSDPFESTRPEQLTRFVGKHFIYTYDNGWQYEMYIKNA
RTIDYRIHSGMVGGRWVRDQEAHIVRLSDDVFKVSWDEPTGTTVSVAVNLAERRLHGV
IFFPQWIAQDPEKTVCFQNDHLDEMRQYRDAGPTYPKLVVDEFATITFVEDCGADDETV
IACAPAELPEGYAARRN

N80

MGSSHHHHHHSSGLVPRGSHMTTVSSPTPTQDLSGIVGKHFIYTYANGWQYEMYVKNA
TTIDYRIHSGMVGGRWVKDQQVDLVRLGEDVYKVSWTEPTGTSVSVNVMPARRRLHG
VIFFPRWVHEHPERTVCFQNDHLDEMRAYRDEGPTYPIHVVSEFAKITFLEDCGPDDETV
IACAPAELPAGYADRTN

N100

MGSSHHHHHHSSGLVPRGSHMTTVENPVPPQDLSGIVGHRFIYTYANGWQYEMYVKN
ATTIDYRIHSGMVGGRWVKDQQVDLVQLDDDSYKVSWNEPTGTSVVVNVMPGKRRL
HGVIFFPRWVEEHGERTVCFQNDHLDQMRAYRDEGPTYPIYVVPEFARITLFEHVGADD
ETVISVAPGDLPAGWADRTN

N122

MGSSHHHHHHSSGLVPRGSHMTAASPPAPDQDLSGIVGKHLIYTYANGWQYELYVKNE
NTIDYRIHSGLVGGRWVKDQPVHIVRLGDGMYKVSWTEPTGTCVSLVVDLAERWLHG
TIFFPQWVSQHPERTVCFQNEHLDEMRAFRDEGPAYPQVVIDEFATITFVEQCGPDDDTV
IDCAPGELPDGYADRTN

N124

MGSSHHHHHHSSGLVPRGSHMPAASHSEPAQDLSGFVGTHFIYTYANGWQYEWYARN
EKTCDYRIHHGLVGGRWVTHQAVHIVRLADGMYKVDWHEPTGTCVSLLFDLPRRLVH
GTIFFPQWIGGEGQHPEKTICYQNEFIDDMHRFRDEGPAYPYVIISEFAKITYMEQRGRDN
DTVIDAAPGQLPDGYADRTN

N134

MGSSHHHHHHSSGLVPRGSHASRPQPDQDLSGIVGKHLIYTYANGWQYELYVKNENTI
DYRIHSGPVGGRWVKDQPVVIVRLGNGMYKVSWTEPTGTCVSLVVDLAERWLHGTIFF
AQWVSQHPELTVVFQNEHLDEMRALRDEGPVYPQVVIDEFATITFVENCGIDNDDVIDC
APGELPDGYIDRTN

N146

MGSSHHHHHHSSGLVPRGSHMAARSTAASSPALAGDLTGIVGKHLIYAYENGWKYELY
VRNSQTIAFRCLMGPMFGRWSTNQSAKIVQLHGDLYKLAWVEPTGTTTVVIAWLSERR
VHTTISYPQWMLDYPESTLGRYEDNLDEIIADRDAGPTYPLTLVASTGRITFLESRAPDD
DTVIDRPPNKLPLGYADRTN

References

(1) Ye, J.; McGinnis, S.; Madden, T. L. BLAST: Improvements for Better Sequence Analysis. *Nucleic Acids Res.* **2006**, *34*, 6–9. https://doi.org/10.1093/nar/gkl164.

(2) Sievers, F.; Higgins, D. G. Clustal Omega for Making Accurate Alignments of Many Protein Sequences. *Protein Sci.* **2018**, *27* (1), 135–145. https://doi.org/10.1002/pro.3290.

(3) Kumar, S.; Stecher, G.; Li, M.; Knyaz, C.; Tamura, K. MEGA X: Molecular Evolutionary Genetics Analysis across Computing Platforms. *Mol. Biol. Evol.* **2018**, *35* (6), 1547–1549. https://doi.org/10.1093/molbev/msy096.

(4) Jones, D. T.; Taylor, W. R.; Thornton, J. M. The Rapid Generation of Mutation Data Matrices. *Bioinformatics* **1992**, *8* (3), 275–282. https://doi.org/10.1093/bioinformatics/8.3.275.

(5) Foley, G.; Mora, A.; Ross, C. M.; Bottoms, S.; Sützl, L.; Lamprecht, M. L.; Zaugg, J.; Essebier, A.; Balderson, B.; Newell, R.; Thomson, R. E. S.; Kobe, B.; Barnard, R. T.; Guddat, L.; Schenk, G.; Carsten, J.; Gumulya, Y.; Rost, B.; Haltrich, D.; Sieber, V.; Gillam, E. M. J.; Bodén, M. *Engineering Indel and Substitution Variants of Diverse and Ancient Enzymes Using Graphical Representation of Ancestral Sequence Predictions (GRASP)*; **2022**; Vol. 18. https://doi.org/10.1371/journal.pcbi.1010633.

(6) Schweiger, A. K.; Ríos-Lombardía, N.; Winkler, C. K.; Schmidt, S.; Morís, F.; Kroutil, W.; González-Sabín, J.; Kourist, R. Using Deep Eutectic Solvents to Overcome Limited Substrate Solubility in the Enzymatic Decarboxylation of Bio-Based Phenolic Acids. *ACS Sustain. Chem. Eng.* **2019**, *16364–1637* (7(19)), 1–19. https://doi.org/10.1021/acssuschemeng.9b03455.

(7) Terholsen, H.; Myrtollari, K.; Larva, M.; Möller, C.; Taden, A.; Kourist, R.; Bornscheuer, U. T.; Kracher, D. Spectrophotometric and Fluorimetric High-Throughput Assays for Phenolic Acid Decarboxylase. *ChemBioChem* **2023**, e202300207. https://doi.org/10.1002/cbic.202300207.

(8) Park, J. S. Muricauda Hymeniacidonis Sp. Nov., Isolated from Sponge of Hymeniacidon Sinapium. *Int. J. Syst. Evol. Microbiol.* **2019**, *69* (12), 3800–3805. https://doi.org/10.1099/ijsem.0.003683.

(9) Chen, W.-M.; Yang, S.-H.; Huang, W.-C.; Cheng, C.-Y.; Sheu, S.-Y. Chitinivorax Tropicus Gen. Nov., Sp. Nov., a Chitinolytic Bacterium Isolated from a Freshwater Lake. *Int. J. Syst. Evol. Microbiol.* **2012**, *62*, 1086–1091. https://doi.org/10.1099/ijs.0.031310-0.

(10) Kloos, W. E.; Zimmerman, R. J.; Smith, R. F. Preliminary Studies on the Characterization and Distribution of Staphylococcus and Micrococcus Species on Animal Skin. *Appl. Environ. Microbiol.* **1976**, *31* (1), 53–59. https://doi.org/10.1128/aem.31.1.53-59.1976.

(11) Goudenège, D.; Labreuche, Y.; Krin, E.; Ansquer, D.; Mangenot, S.; Calteau, A.; Médigue, C.; Mazel, D.; Polz, M. F.; Le Roux, F. Comparative Genomics of Pathogenic Lineages of Vibrio Nigripulchritudo Identifies Virulence-Associated Traits. *ISME J.* **2013**, *7* (10), 1985–1996. https://doi.org/10.1038/ismej.2013.90.

(12) Jumper, J.; Evans, R.; Pritzel, A.; Green, T.; Figurnov, M.; Ronneberger, O.; Tunyasuvunakool, K.; Bates, R.; Žídek, A.; Potapenko, A.; Bridgland, A.; Meyer, C.; Kohl, S. A. A.; Ballard, A. J.; Cowie, A.; Romera-Paredes, B.; Nikolov, S.; Jain, R.; Adler, J.; Back, T.; Petersen, S.; Reiman, D.; Clancy, E.; Zielinski, M.; Steinegger, M.; Pacholska, M.; Berghammer, T.; Bodenstein, S.; Silver, D.; Vinyals, O.; Senior, A. W.; Kavukcuoglu, K.; Kohli, P.; Hassabis, D. Highly Accurate Protein Structure Prediction with AlphaFold. *Nature* **2021**, *596* (7873), 583–589. https://doi.org/10.1038/s41586-021-03819-2.

(13) Bowers, K. J.; Chow, E.; Xu, H.; Dror, R. O.; Eastwood, M. P.; Gregersen, B. A.; Klepeis, J. L.; Kolossvary, I.; Moraes, M. A.; Sacerdoti, F. D.; Salmon, J. K.; Shan, Y.; Shaw, D. E. Scalable Algorithms for Molecular Dynamics Simulations on Commodity Clusters. *Proc. 2006 ACMIEEE Conf. Supercomput. SC06* **2006**, No. January. https://doi.org/10.1145/1188455.1188544.

(14) Baker, E. N.; Hubbard, R. E. Hydrogen Bonding in Globular Proteins. *Prog. Biophys. Mol. Biol.* **1984**, *44* (2), 97–179. https://doi.org/10.1016/0079-6107(84)90007-5.

(15) Kathuria, S. V.; Chan, Y. H.; Nobrega, R. P.; Özen, A.; Matthews, C. R. Clusters of Isoleucine, Leucine, and Valine Side Chains Define Cores of Stability in High-Energy States of Globular Proteins: Sequence Determinants of Structure and Stability. *Protein Sci.* **2016**, *25* (3), 662–675. https://doi.org/10.1002/pro.2860.

(16) Strickler, S. S.; Gribenko, A. V.; Gribenko, A. V.; Keiffer, T. R.; Tomlinson, J.; Reihle, T.; Loladze, V. V.; Makhatadze, G. I. Protein Stability and Surface Electrostatics: A Charged Relationship. *Biochemistry* **2006**, *45* (9), 2761–2766. https://doi.org/10.1021/bi0600143.

(17) Sternke, M.; Tripp, K. W.; Barrick, D. Surface Residues and Nonadditive Interactions Stabilize a Consensus Homeodomain Protein. *Biophys. J.* **2021**, *120* (23), 5267–5278. https://doi.org/10.1016/j.bpj.2021.10.035.

(18) Mirdita, M.; Schütze, K.; Moriwaki, Y.; Heo, L.; Ovchinnikov, S.; Steinegger, M. ColabFold: Making Protein Folding Accessible to All. *Nat. Methods* **2022**, *19* (6), 679–682. https://doi.org/10.1038/s41592-022-01488-1.

(19) Molinaro, A. M.; Simon, R.; Pfeiffer, R. M. Prediction Error Estimation: A Comparison of Resampling Methods. *Bioinformatics* **2005**, *21* (15), 3301–3307. https://doi.org/10.1093/bioinformatics/bti499.

(20) Barupal, D. K.; Fiehn, O. Generating the Blood Exposome Database Using a Comprehensive Text Mining and Database Fusion Approach. *Environ. Health Perspect.* **2019**, *127* (9), 2825–2830. https://doi.org/10.1289/EHP4713.

(21) Pucci, F.; Kwasigroch, J. M.; Rooman, M. SCooP: An Accurate and Fast Predictor of Protein Stability Curves as a Function of Temperature. *Bioinforma. Oxf. Engl.* **2017**, *33* (21), 3415–3422. https://doi.org/10.1093/bioinformatics/btx417.

(22) Evans, P. Scaling and Assessment of Data Quality. *Acta Crystallogr. D Biol. Crystallogr.* **2006**, *62* (1), 72–82. https://doi.org/10.1107/S0907444905036693.

(23) Evans, P. R. An Introduction to Data Reduction: Space-Group Determination, Scaling and Intensity Statistics. *Acta Crystallogr. D Biol. Crystallogr.* **2011**, *67* (4), 282–292. https://doi.org/10.1107/S090744491003982X.

(24) Evans, P. R.; Murshudov, G. N. An Introduction to Data Reduction: Space-Group Determination, Scaling and Intensity Statistics. *Acta Crystallogr. D Biol. Crystallogr.* **2013**, *62* (7), 1204–1214. https://doi.org/10.1107/S0907444905036693.

(25) Krissinel, E.; Uski, V.; Lebedev, A.; Winn, M.; Ballard, C. Distributed Computing for Macromolecular Crystallography. *Acta Crystallogr. Sect. Struct. Biol.* **2018**, *74*, 143–151. https://doi.org/10.1107/S2059798317014565.

(26) Winn, M. D.; Ballard, C. C.; Cowtan, K. D.; Dodson, E. J.; Emsley, P.; Evans, P. R.; Keegan, R. M.; Krissinel, E. B.; Leslie, A. G. W.; McCoy, A.; McNicholas, S. J.; Murshudov, G. N.; Pannu, N. S.; Potterton, E. A.; Powell, H. R.; Read, R. J.; Vagin, A.; Wilson, K. S. Overview of the CCP4 Suite and Current Developments. *Acta Crystallogr. D Biol. Crystallogr.* **2011**, *67* (4), 235–242. https://doi.org/10.1107/S0907444910045749.

(27) McCoy, A. J.; Grosse-Kunstleve, R. W.; Adams, P. D.; Winn, M. D.; Storoni, L. C.; Read, R. J. Phaser Crystallographic Software. *J. Appl. Crystallogr.* **2007**, *40* (4), 658–674. https://doi.org/10.1107/S0021889807021206.

(28) Murshudov, G. N.; Skubák, P.; Lebedev, A. A.; Pannu, N. S.; Steiner, R. A.; Nicholls, R. A.; Winn, M. D.; Long, F.; Vagin, A. A. REFMAC5 for the Refinement of Macromolecular Crystal Structures. *Acta Crystallogr. D Biol. Crystallogr.* **2011**, *67* (4), 355–367. https://doi.org/10.1107/S0907444911001314.

(29) Emsley, P.; Lohkamp, B.; Scott, W. G.; Cowtan, K. Features and Development of Coot. *Acta Crystallogr. D Biol. Crystallogr.* **2010**, *66* (4), 486–501. https://doi.org/10.1107/S0907444910007493.

(30) Corso, G.; Stärk, H.; Jing, B.; Barzilay, R.; Jaakkola, T. DiffDock: Diffusion Steps, Twists, and Turns for Molecular Docking. **2022**. https://doi.org/10.48550/arXiv.2210.01776.
